# Application of an adapted FMEA framework for robot-inclusivity of built environments

**DOI:** 10.1038/s41598-022-06902-4

**Published:** 2022-03-01

**Authors:** Y. J. Ng, Matthew S. K. Yeo, Q. B. Ng, Michael Budig, M. A. Viraj J. Muthugala, S. M. Bhagya P. Samarakoon, R. E. Mohan

**Affiliations:** 1 Singapore University of Technology and Design, Engineering and Product Development, Singapore, 487372 Singapore; 2 Singapore University of Technology and Design, Architecture and Sustainable Design, Singapore, 487372 Singapore

**Keywords:** Civil engineering, Sustainability

## Abstract

Mobile robots are deployed in the built environment at increasing rates. However, lack of considerations for a robot-inclusive planning has led to physical spaces that would potentially pose hazards to robots, and contribute to an overall productivity decline for mobile service robots. This research proposes the use of an adapted Failure Mode and Effects Analysis (FMEA) as a structured tool to evaluate a building’s level of robot-inclusivity and safety for service robot deployments. This Robot-Inclusive FMEA (RIFMEA) framework, is used to identify failures in the built environment that compromise the workflow of service robots, assess their effects and causes, and provide recommended actions to alleviate these problems. The method was supported with a case study of deploying telepresence robots in a university campus. The study concluded that common failures were related to poor furniture design, a lack of clearance and hazard indicators, and sub-optimal interior planning.

## Introduction

There is burgeoning use of Artifical Intelligence (AI), Information and Communication Technologies (ICT) and service robots as cities transition towards “Smart Cities”. Productivity increase^1^, cost reduction^2^ and reduced reliance on human labour^3^ are some of the wide ranging benefits that implementation of service robots and AI bring to organisations across various industries. Often defined as a physical embodiment of a computer system with a certain level of autonomy^2,4^, service robots aid in a wide spectrum of use applications such as healthcare, manufacturing and education. Delivery services on mobile robotic platforms, teachers operating as avatars and humans meeting remotely for training and education are just some of the possibilities of applications identified in^5^.

In the setting of smart campuses, Dong et al.^6^ highlighted that emerging technologies can be integrated to provide new learning opportunities such as via virtual systems to interact in cyber-physical conditions. For mobile robotic systems, data inputs from sensory information obtained from the environment are used in machine learning and map-building^7^ are crucial elements for autonomous navigation or inducing other actions^8^. These robots are often equipped with various sensors and technical algorithms such as Simultaneous Localization and Mapping (SLAM) to sense their surroundings, localise and to move. It is thus essential that the built environment supports the leveraging of such advanced technology in smart campuses and smart cities.

In order for humans to reap the full benefits of utilising service robots, it is vital to consider and ensure the safety of these service robots^9^. This brings up the importance of clarifying the definition of the term “robot safety” to gain a better understanding of who the target stakeholders are in the aspect of human-robot interactions (HRI). Dhillon^10^ outlined three aspects of robot safety: preventing damage on the environment by robots, preventing harm to humans by robots, and preventing damage to the robots themselves. The term “robot safety” in this paper refers to the safety of service robots. Ensuring the safety of robots will allow the robot to perform at its full potential where the completion of required tasks are guaranteed and to achieve high productivity rates in the process.

Most works define the term “robot safety” as safety to humans^11–13^ during their operation. Like any other machinery under normal circumstances, human accidents or injuries can happen due to factors such as the failure of a robot’s components^14^, unpredictable movements^15^ by both robots and humans, as well as the crossing of workspace boundaries^16^ between the two. In most cases, the problem is mitigated through enhancing the robot’s hardware and software, exploring deployments of advanced controlling methods^17,18^, complex hardware designs^19^, robust sensors integration^20,21^, reconfigurable mechanisms^22^, and artificial intelligence frameworks^23^. Under HRI, other methods of ensuring safety of robots to humans include safety-rated monitored stop, hand-guiding, speed and separation monitoring, power and force limiting^24–26^. Employing these methods improve safety by targeting mainly aspects of collision avoidance, failure prevention or human contact safety^27,28^.

On the other hand, there is less consideration in ensuring the robot safety in terms of preventing damage to the robot. Damages to the robot could be a result of environmental factors or hazards^29^. Just as how sudden changes in elevation or obscure protrusions can be potential causes for human injury, robots are also subjected to possible damage from such hazards. While service robots are equipped with different levels of autonomy to sense their surroundings to carry out tasks that require interaction with their environment such as cleaning, building or transporting, they face different environmental conditions that exert varying degrees of difficulties and complexities on them^1^. Unstructured environments call for highly complex and robust navigational and obstacle avoidance systems within the robot to adapt to changes in the environment^28^. These issues are not usually anticipated during the design development of the robots. This means that the robots are expected to operate within “environments that have not been pre-prepared for [their] operation^27^”. As a result, robots are unable to perform their expected tasks or to complete them in an efficient manner, defeating the purpose of their deployments in the first place. A reduction of challenges in the built environment would in turn decrease the complexity required in the robotic control system, enabling more affordable and robust robots.

While some papers have studied the hazards within the operating environments of the robot^27,28^, there have not been any studies on looking into the safety of buildings as a whole for robots. Considering how robots, like humans, are also occupants of a building, there is a cause for studying the safety in built environments for robots in a systematic manner, analysing buildings by their various components with the greater purpose of improving the built infrastructure for robots. Ultimately, the efficient deployment of service robots requires the mutual integration of both the robot functionality and the working environment^30^.

In this regard, a common hazard identification system method known as the Failure Mode and Effects Analysis (FMEA)^31–33^ is considered for determining hazards faced by the service robots during their operation in their respective environments. By incorporating environments in buildings and their respective components as objects of interest in the FMEA approach, this paper proposes an adapted FMEA framework that is made to be robot-inclusive (RIFMEA). It can be used for ascertaining hazards for service robots in various built environments, to improve the safety of robots for humans, for robots as well as for the environment. In this paper, a case study application of the RIFMEA is carried out through the deployment of telepresence robots in the Singapore University of Technology and Design (SUTD) campus. Hazards for robots within their workspaces are identified, categorised and tagged to the relevant building components and elements, before being analysed to assess its cause and consequence on the robot’s operation.

The key objective behind hazard identification and classification is to improve the design of buildings with spatial recommendations in order to minimise failures and risks imposed by the environment on the robot. By making it easier for robots to carry out their tasks, more desirable interactions and cooperation between humans and robots can be achieved^34^. This will also contribute to minimising the wear and tear of the robot, failure rates and maintenance costs^35^, ultimately boosting productivity of robotic deployments for strenuous and menial tasks.

In the following sections, we evaluate the results of the test runs of an application of the proposed RIFMEA in which the Double 3 telepresence robot 3 was deployed in different environments within a university campus.

## Results

The RIFMEA considers the parameters of Severity (S), Occurrence (O), and Detection (D), which are ratings tagged to each failure mode to compute the Risk Priority Number (RPN) for the corresponding failure. The RPN value is calculated by multiplying the S, O and D ratings together whereby RPN=S*O*D. The RPN, which ranges from 1 to 125 for our proposed rating structure, is a means to rank failure modes in terms of the need for corrective actions to prevent or reduce the probability of the failure occurring^33^. More details on the individual rating systems are elaborated within the “Modified S,O,D Rating System” subsection found in the later sections below.

Three diagnostic tests and two task-based tests were conducted where the Double 3 telepresence robot was deployed across four different locations within the Singapore University of Technology and Design (SUTD) campus to gather data required for the proposed RIFMEA method. The test sites are shown in Fig. 4. Within the tests, building components and their elements the robots interacted with when failures occurred were identified. As seen in Fig. 2B, the building components are divided into five categories: Structure, Architecture Exterior, Architecture Interior, Services and Plan. The descriptions of the experiments and a breakdown of the proposed RIFMEA methodology are elaborated within the “Methods” section of the paper.

The Double 3 robot used for the study is intended for telepresence tasks, such as remote navigation and communication which requires a robot operator to perform. On the interface screen which shows the teleoperator what the robot sees through its camera in real time, there is an overlay of a grid of dots which denotes the accessible areas of the robot. For navigation, the teleoperator initiates high-level commands by marking a goal point on the accessible areas as the teleoperator views the scene through the cameras on the robot. Moreover, the operation of the robot is semi-autonomous; the user does not need to concentrate on low-level functions such as providing continuous velocity and steering commands. The teleoperator selects a location, and the robot itself performs obstacle avoidance and path planning to navigate towards the given goal. In addition, the robot can also be moved by using the arrow keys on the computer keyboard.

The type of hazards or failures identified are largely influenced by the exact sensing and detection abilities of the robot, which in turn depend on the sensors adopted for perceiving the environment. Most present-day robots rely on sensors such as lidars and vision systems. However, information from multiple sensors is fused to further improve reliability and accuracy. Even though different sensors are available, the characteristics of sensors can be grouped. For example, 2D/3D lidars and other IR-based proximity sensors can fail in detecting glass walls since the IR penetrates through such materials without reflecting. Vision-based systems could also experience the same issue. On the other hand, sensors such as ultrasonic are good at detecting transparent materials, although they may fail in situations where the reflection characteristics are poor. The Double 3 robot used for this study is equipped with both stereovision depth sensors and ultrasonic range sensors for perceiving the environment. Therefore, the hazards and failures in this study would be relevant to these two major groups of sensing technologies commonly used by most of the present-day robots.

The Double 3 robot has an adjustable neck to control its height and has various operating speed modes to choose from. However, the detectability of objects could possibly be influenced by the height at which the sensors are placed, which would affect their effective range, as well as the speed at which the robot approaches the objects due to the communication and response time required by its sensors. As such, to minimise error deviations stemming from these factors, we have kept the maximum speed and height of the robot constant throughout the experiments.

### Failures across test sites

**Figure 1:**
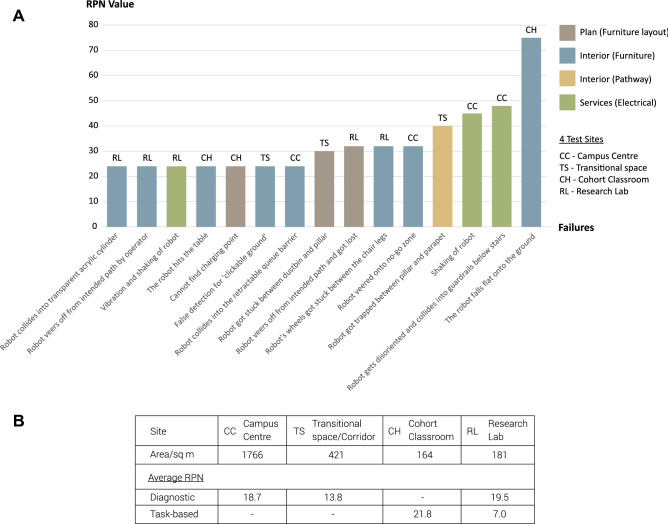
Summary of findings from test runs. **(A)** Failures with highest RPN values. **(B)** Table of Average RPN values for diagnostic and task-based tests.

The top 15 failures with the highest RPN values out of the total 65 failures are seen in Fig. 1A. The failure counts were based on the total number of times a failure occurred when the robot interacted with any building component at any one time. Evidently, based on Fig. 1A, the elements in buildings that contributed to these failure modes were mainly poor furniture design, followed by sub-optimal furniture layout, the presence of electrical casings and outlets on the ground, as well as the lack of spatial clearance. As a whole, the mismatch between the ability of the robot to detect and sense the furniture contributed to 25 out of 65 failure counts in total. 9 out of 25 of furniture-related failures were in relation to office chairs, highlighting that the fact that the design of the office chair in particular was a problem for the robot. The cause of the failures were largely attributed to the limited ability of the 3D sensors to detect slender, linear objects. As a result, the robot either got stuck between the chair legs at times, or the robot veered off course from its intended path, causing inefficiencies.

The failure with the highest RPN value of 75 was identified in the Cohort Classroom, which is a space used for project-based learning and allows for flexible furniture layouts (Fig. 4C). The failure was observed when the robot fell flat onto the ground and caused sustained damage to its components. The robot was disengaging its retractable brakes while it was very close to a table. The cause of the failure mode was partly due to the self-balancing feature of the robot and the limited ability of the 3D sensors to detect the linear form of the table top edge. A disconnection issue during the event further exacerbated the situation.

A comparison of the average RPN values of all four zones in the building—the Cohort Classroom, a transitional space, a research lab and the Campus Centre was made, as shown in Fig. 1B. Comparison studies of task-based tests and diagnostic tests with references to the failures identified in Fig. 1B are discussed further in the sections below.

### Task-based run: Cohort Classroom and research lab

The average RPN for the task-based test run in the research lab (Fig. 4D) was 7.0 as compared to 21.8 for the Cohort Classroom. The higher average RPN value for the classroom was due to the serious failure mode in which the robot fell flat onto the ground. The failure attained a rating of 5 for both the Severity and Detection ratings, which raised the average RPN value for the Cohort Classroom findings.

As compared to the Cohort Classroom, the research lab had a very low average RPN value of 7.0 in for its task-based run. The task-based study conducted within the research lab yielded only 3 failure modes stemming from poor furniture layout. The failure modes arose due to the lack of clearance imposed by the furniture that were loosely placed within the environment. As a result, the robot was unable to smoothly circumvent and navigate through the obstacles due to the tight pathways, causing inefficiencies in recalibration and reorientation to continue moving. In all of the failures, the Severity rating had a low value of 1. As the space within the lab was more cluttered as a result of cluttered furniture placement, the study generated more failure modes relating to the Plan component as compared to the study within the classroom.

The task-based study in the Cohort Classroom also revealed an interesting failure mode—the inability of the remote user to locate the charging point. This failure relates to the furniture layout of the classroom setting. The spatial layout or features were not informative and intuitive enough for remote users to know and identify where the charging point was located. This was due to lack of peripheral visual cues as well as the poor line of sight of the floor area through the lens of the robot for the remote user, resulting in a high Detection score.

In general, it was expected that the task-based runs would reveal lesser failure modes than in the diagnostic runs, since the total area covered by the robot is reduced for task-based runs. However, the runs shed light on some interesting failure modes, which were not observed when carrying out the diagnostic tests.

### Diagnostic tests: transitional space, research lab and Campus Centre

The highest average RPN value among the diagnostic tests were found to be from the test performed in the research lab. Given that the research lab had the smallest area of only 181 square metres, the cluttered spatial layout made it difficult for the robot to navigate, contributing to several failure modes whereby the robot got stuck between obstacles or tight paths. Furthermore, the office chairs were supported on multi-pronged legs that were too low to the ground, which resulted in conflicting signals received by the robot sensors as irregular surface depths were detected. This led to inefficiencies from the robot, expending time and power in recalibration and reorientation. Most of the failures recorded were mainly due to the lack of clearance in the pathway and the ill-suited design of office chairs.

Within the transitional space (Fig. 4B), the failure modes that presented the highest RPN values were attributed to the Interior building components and furniture layout. In these cases, the robot was either rendered immobile or took an extended amount of time to recalibrate and reorientate itself. Origins of these failure modes were found to be tight and narrow spaces, causing accessibility issues. The failure mode that produced the highest RPN value of 40 occurred when the robot moved into a narrow but accessible space between a structural column and a glass parapet. As the robot approached the tight corridor, the proximity sensors and collision avoidance algorithm of the robot caused the robot to rotate into a position which eventually rendered the robot completely immobile as the conflicting sensor data prevented the robot making any further movements. Hence, the robot had to be rescued by external intervention. While this failure is unlikely to occur under a typical use case setting, running the diagnostic test allowed us to identify such a potential hazard. This also highlighted the potential problems of tight or narrow spaces that are just wide enough for the robot to pass through without giving any clearance.

The Campus Centre (Fig. 4A) is a multi-purpose, double-storey high, central lobby of the campus that is regularly used for public events or exhibitions. Here, the failure modes that presented the highest RPN values were attributed to the Interior building component. Often the failures arose from the difficulty in detecting various types of furniture. Portions of a permanent exhibit structure, known as the Time Capsule, as well as furniture fittings such as the sofa were out of the robot’s sensor range as they were located either too high or too low. However, failures that produced the higher RPN rating came from the Services building component. In particular, the cables on the floor contributed to these failures. The high RPN values were due to the high Occurrence and Detection rating. Although loose cables are additions and do not necessarily form part of the building, cable trunking or covers affixed onto the ground are more permanent components, acting as bumps on the ground which are a cause of failures for the robot. In our test run, the cable casing cover induced a sudden directional change when the robot tried approaching it at an angle. This caused the robot to hit against a metal railing guard located next to the cable casing before the robot could respond in time to the sudden induced direction change.

Besides the failures with the highest RPN values, it is also worth mentioning other failures that were outliers or extreme cases that had a score of 5 in any of the S,O,D categories. In the diagnostic test conducted in the transitional space, the robot fell into a no-go zone when it was operating very close to a staircase for an extended period of time. The failure was due to a misalignment of the 3D vision sensors when the robot tried to rotate and move forward. The failure mode led to a Severity rating of 5. While the Occurrence and Detection rating for this failure were low, it may be a cause for concern in the event that a robot would end up moving too close to the edge of a staircase, given that the robot did fall onto the stairs during our diagnostic test run despite its cliff detection sensors being effective and responsive most of the time.

In the same test run within the transitional space, the robot falsely detected navigable ground when viewing closed transparent doors. This implies that the robot had falsely identified an accessible ground plane in which the robot could travel on. The failure mode occurred to all doors the robot interacted with in the space, which led to an Occurrence rating of 5. The 3D vision sensors of the robot could not detect the transparent glass surfaces and did not register glass surfaces as obstacles. This failure could potentially cause problems in the path planning process of the robot if what the robot perceives in its surrounding is inaccurate. It is therefore crucial to consider the materiality and finishing of building elements in improving the sensing of obstacles by the robot.

Another failure mode with a Detection rating of 5 was the hitting of robot against the door stopper affixed on the ground. Even though the Occurrence rating was low as it was located very close to the wall, the door stopper was very difficult to detect due to its small size and it being very low on the ground. As such, the presence of other similar obstacles outside the range of the robot’s vision may similarly have the potential to cause wear and tear of the robot, shortening its shelf life.

## Discussion

An extract of the RIFMEA worksheet of consolidated failures for all four test areas is shown in Table 1 while the full records of the tests are attached as supplementary material to this paper. During the experiments, there exists a challenge in determining the exact cause of failure or the failure mode itself as it is not often very clear. For example, during our tests, we observed that transparent surfaces such as glass railings or full-height glass doors were often incorrectly shown as accessible regions on the robot’s interface screen. While the ultrasonic sensors on the robot are supposed to detect transparent materials, the information from these sensors did not appear to be translated into the interface screen and users were allowed to designate these areas as goal points. Despite this, we observe that the robot could still autonomously avoid colliding into such surfaces when coming in close proximity, showing that the ultrasonic sensors were able to take effect. These failures were thus noted down as “false detection for ’clickable ground’ ”.

We also observed that the robot has limitations with regards to being able to accurately sense whether a particular path has sufficient spatial clearance for it to pass or not. Although some narrow pathways were shown up as accessible areas, upon approach, the robot failed to navigate through them due to the sensing of surrounding obstacles, entering into a constant loop of approaching and avoiding. We can thus see a discrepancy between the robot’s sensor feedback and its accessible area reflected by the interface screen. In these cases, we concluded that insufficient clearance is the main cause to these inefficiencies.

Despite having autonomous obstacle detection and avoidance capabilities, there are also other instances during navigation where the Double robot still collides with objects, often those with thin, linear-like profiles such as office chairs. In addition, we observed that the robot sometimes makes sudden path changes when coming in close proximity with such objects, revealing inconsistencies in the detection sensors. We thus concluded that the sensors on the robot have difficulty in accurately detecting these profiles.

From our studies, a major contributing factor to failure modes was the lack of spatial clearance. The failures occurred when the robot was made to navigate through tight spaces, which were often caused by a sub-optimal furniture layout as observed in the research lab and the lift lobby area on level 6. Such problems were absent in the campus centre where it was more spacious. As a general rule, accessible corridor spaces for the robot should have a minimum width clearance of about 10cm on both sides of the robot’s body, as well as about 10cm height clearance to cater for the robot’s head, and provide for the robot’s turning radius. Narrow spaces which are dangerous for the robot to enter should have their boundaries demarcated clearly by providing more hazard indicators (be it physical or virtual) to warn the teleoperator of possible dangers and preventing the operator from moving the robot over these zones. Recommended changes to furniture layouts would involve rearranging the movable fittings such that it allows for sufficient clearance for robot to pass through, and thus allow the robot to reach the zones it is meant to access during operation.

Glass surfaces and slender, linear-shaped objects were also found to be causes for several failure modes. Built-in structures like glass parapets and glass doors were a challenge for the robot sensors to detect as obstacles, as well as other movable furniture such as signages or office chairs. In addition, thin horizontal elements like the tabletop edges were also hard-to-detect objects for the robots. In these cases, increasing the detectability of the obstacles by the robot’s sensors would be key to reducing possible collisions or other inefficiencies. For glass, design changes to the surface such as fritted patterns, frosted or opaque finishes would help increase detectability of the glass by the 3D vision sensors on the robot. While changing the design of slender and linear elements to a bulkier form would help increase detectability by the robot’s sensors, it would not be an ideal solution when considering aesthetics. Much thought would need to be given to the furniture designs, to strike a balance between a design that is aesthetically pleasing, yet easy to detect for the robot. Another potential solution would be to add visual demarcations to prevent the teleoperator from driving the robot too close to such hazards.

Bumps on the ground surface caused by electrical components also contributed to the failure modes, causing shaking of the robot when the robot navigates over them. This includes the cables and electrical power boxes on the ground, which are not entirely flushed with the ground surface. Other protrusions on the ground include the door stoppers which are fixed to the ground. These obstacles are very close to the ground and are difficult elements for the robot to detect. While one solution may be to avoid going across these obstacles completely by rerouting the wiring, this method can cause large infrastructural inefficiencies. Reworking loose electrical cables to house them within the flooring would be ideal but can result in drastic changes to the existing or future building structure. Where cables cannot be relocated, cable trunking or covers should be used but they should not compromise the accessibility of both humans and robots and should allow for both wheelchair users or robots to travel over them easily. Introducing a more wheel-friendly casing or trunking design for the cables might be a more feasible solution.

The main recommended actions in response to the above failures largely involve the rearranging of space, furniture and layouts to allow for sufficient clearance to maximise the robot’s accessibility. Provision of more hazard indicators to improve detection for both robot and the operator would also be important. Fitting the deployment area with furniture that can be easily detected by the robot would be important in improving the robot’s observability. Some of the solutions are more context-dependent and specific such as providing visual and wayfinding cues for the location of the charging dock. While some solutions may not be easily implemented or require more large-scale changes, they are recorded for reference for future building designs and layouts. These recommendations would also be listed as follow-up actions to do on the RIFMEA worksheet as well.

**Table 1:** Extract of consolidated RIFMEA findings from all test sites.

Building	Zone	Building component	Building element	Robot-inclusive principles		Failure	Effect	Severity[S]	Cause	Occurrence[O]	Detection[D]	RPN (S*O*D)	Recommended action
Building 2	Level 6 Research lab	Plan	Furniture layout	Activity	Observability	Robot veers off from intended path and got lost	Inefficiency due to extended recalibration and reorientation	2	Too many obstacles in close proximity, causing interference with sensors	4	4	32	Adjust movable obstacles to provide clearance
		Interior	Furniture (transparent acrylic cylinder)	Observability		Robot collides into transparent acrylic cylinder	Safety risk for robot	2	3D Vision camera cannot detect transparent surfaces well, Sonar sensors could not detect the curved surface of the item	4	3	24	Improve obstacle detectability through additions of visual markers
			Furniture(TV stand)	Observability	Accessibility	Robot veers off from intended path by operator	Inefficiency due to extended recalibration and reorientation	3	Linear and point objects are difficult to detect from a safe distance	2	4	24	Consider alternative design for the TV stand base
			Furniture (Office chair)	Observability	Accessibility	Robot’s wheels got stuck between the chair legs	Inefficiency due to extended recalibration and reorientation	2	Obstacle is too low and out of sensor’s range to be detected at a safe distance	4	4	32	Consider alternative chair design
		Services	Electrical outlet cover	Accessibility	Observability	Vibration and shaking of robot	Minor damage to robot, Inefficiency due to extended recalibration and reorientation	2	Box with cable spacers that protrude above the ground	4	3	24	Demarcate no-go zones by placing markers around electrical outlet
	Level 6 Cohort Classroom	Interior	Furniture (table)	Observability		The robot hits the table	Damage to robot and table	2	Sensors were unable to detect table as obstacle	3	4	24	Provide markings around table to denote safe, accessible areas/boundaries
				Observability	Manipulability	The robot falls flat onto the ground	Damage to robot and table, damage to robot casing and self-balancing required recalibration	5	The robot’s self balancing mechanism conflicted with its obstacle detection capabilities when unparking	3	5	75	Provision of stable internet network. Provide markings around table to denote safe, accessible areas/boundaries
		Plan	Furniture layout	Activity	Observability	Cannot find charging point	Inefficiency due to reorientation	2	User did not have information about surrounding whereabouts	3	4	24	Provide visual/wayfinding markers to denote location of charging dock
Building 2,3	Level 6 Transitional space	Interior	Furniture (Cantilevered table)	Observability		False detection for ’clickable ground’	Minor damage to robot and environment, Inefficiency due to extended recalibration and reorientation	2	Detectable area of the table for the robot is too limited	4	3	24	Improve obstacle detectability through additions or introduce visual demarcations on table
			Pathway (gap between parapet and pillar)	Accessibility		Robot got trapped between pillar and parapet	Inefficiency due to extended recalibration and reorientation	2	The width of pathway just fits robot’s width, proximity sensor detects as insufficient clearance	5	4	40	Provide visual demarcations on safe distance away from the pillar
		Plan	Furniture layout	Accessibility		Robot got stuck between dustbin and pillar	Inefficiency due to extended recalibration and reorientation	2	The width of pathway just fits robot’s width, proximity sensor detects as insufficient clearance	5	3	30	Reposition dustbin
	Level 1 Campus Centre	Interior	Furniture (Retractable queue barrier)	Observability		Robot collides into the retractable queue barrier	Possible damage to robot and surroundings	2	Difficulty of robot in detecting linear objects at particular height	3	4	24	Consider alternative types of queue barriers that allows easy detectability
			Furniture (Sofa)	Activity	Observability	Robot veered onto no-go zone	Contribute to wear and tear of robot. Disorientation for operator. Possible damage to robot and surroundings	4	Sofa cushion surface cannot be differentiated with the ground surface. Cannot detect steep level change near sofa	2	4	32	Demarcate no-go zones. Add cushioning around edges to prevent robot entry
		Services	Cable casing	Accessibility	Observability	Robot gets disoriented and collides into guardrails below stairs	Safety risk for robot	4	Robot wheels overcame cable casing bump near guardrails and completely lost balance	3	4	48	Rewire trunking/casing to an area away from the metal railing. Otherwise, introduce a gentler gradient
			Cables on floor	Accessibility		Shaking of robot	Contribute to wear and tear of robot. Disorientation for operator	3	Loose cables acting as bumps for the robot	5	3	45	Add trunking/casing with a gentler gradient

## Prior research and adapted FMEA approach

### Related industry standards

There have been developments of international safety standards such as the ISO 10218^36^or ISO 13482^37^detailing the safety requirements of various robots categories and the need to perform risk assessments for the robots. However these guidelines are largely geared towards reducing possible risks caused by the robots to humans or to their surroundings. There is seldom a consideration for safety issues or hazards posed by the environments the robots operate in found within these standards^38^.

ISO 10218^36^ is a standard on industrial robot safety requirements. While the safety principles within can be applied to robots and industrial robotic manipulators, the standard deals mainly with the robot design and construction and its installation within facilities. The safety measures listed include stopping functions and power and force limiting requirements focused on the robot’s end^39^. Operating methods in collaborative applications were introduced but also dealt largely with control and monitoring systems with little focus on the built environment. Similarly, hazards identified in the standard such as mechanical, thermal and electrical hazards generally pertain to the build of the robot^40^.

The ISO 13482^36^ is described as the sole reference standard for personal care robots^41^, detailing safety requirements for mobile servant robots, physical assistant robots, person carrier robots in non-industrial environments. Safety performance standards are specified for assessment and certification, as well as risk analysis and reduction of the 22 hazard types listed within the standard, which includes categories such as hazardous autonomous action and hazards due to robot start-up^42,43^. However, there is a larger emphasis on the performance level, design, and safety validation on the robot^44^. There is also a lack of test methods for each safety section^42^. Also, while standards such as ISO 18646^45^and ISO 23482^46^ define evaluation methods for mobile service robot performance^47^, they are mainly focused on assessing the robot’s ability with little emphasis on the impediments towards robots caused by the surroundings.

The RIFMEA builds upon these safety guidelines by providing a standardised way to explicitly draw out the spatial hazards within the robot’s working environment as well as proposing a rating system to assess the hazards. Building owners can then prioritise these hazards in terms of the danger posed onto the robot, taking steps to rectify them. The RIFMEA thus complements the available standards by capturing the robot hazards from a different perspective of the building elements and their interaction with the robots.

### Failure mode and effects analysis (FMEA)

Our proposed framework is based on the FMEA approach^33^, which is a systematic method by which a product’s components or process is examined to identify potential failures, their causes and subsequent effects on the system. While FMEA is often applied to products or processes, this paper would scrutinise the building where the service robot is deployed in instead. FMEA is an inductive reasoning process to identify and understand the potential failures of each components of the building in hope of reducing and minimising safety accidents from occurring.

The FMEA procedure assigns a numerical value to each identified failure, using Severity (S), Occurrence (O) and Detection (D) as metrics. The Severity metric refers to the magnitude or gravity of the consequence of a system failure. The more severe the consequence, the higher the value of severity that is assigned to the failure. The Occurrence metric refers to the frequency that a failure is likely to occur. The greater the frequency the failure occurs, the higher the Occurrence rating. The Detection metric refers to the qualitative likelihood of detecting the failure before it occurs to preempt the failure from happening in the first place. The harder it is to detect the failure before it happens, the greater the Detection rating tagged to it.

When carrying out the FMEA, ratings of S, O, D are assigned to each failure and are multiplied to give a Risk Priority Number (RPN), where RPN=S*O*D. The RPNs are then ordered to create a priority list of failures to resolve. Different rating systems are used across various FMEA efforts, with some using alphabetical gradings, and others using numerical grading systems.

### Literature review of applications of existing FMEA framework

The FMEA approach has been applied in existing research work to assess the failures of building designs or building components. In^48^, Yang et al. looked into isolating and prognosticating faults in the heating, ventilation and air-conditioning (HVAC) systems of buildings. Machado et al.^49^ assessed the accessibility of faculty buildings in a university, accounting for Persons with Disabilities (PWD). The work^50^ studied an adapted FMEA framework to identify, categorise and prioritise the latent safety threats of newly constructed buildings for human users while the study^51^ applied an approach based on the FMEA to assess risks and failures in a large-scale building project delivery. However, there are yet to be papers that discuss how the FMEA approach can be adopted to assess building designs on ensuring the safety of robotic deployments.

Works sharing a similar thought trajectory to this paper include^27^ and^28^, which highlighted the need to identify hazards beyond the robot’s intended necessary functions to allow the robot to maintain its state of operational readiness. In both works, they assessed the area of operation and evaluated the spatial conditions and quality through the lens of the service robots, and recognising them, as important stakeholders in the equation in addition to humans. In^27^, Dogramadzi et al. proposed a new variant of hazard analysis known as the Environmental Survey Hazard Analysis (ESHA) approach, classifying potential hazards for autonomous mobile robots into 3 categories: Environmental Features, Objects and Agents. While the method provided a helpful classification framework to analyze potential environmental-related threats, the paper raised concerns over their application being a relatively shallow breadth-first approach. It also highlighted the challenge in choosing a hazard classification scheme that would provide full coverage of all the possible non-mission interactions that take place in any robotic application.

In contrast, the FMEA approach may provide a more comprehensive framework to better identify and categorise the causes of failures, whether it is a failure on the robot’s part or in the design of the environment. In that sense, the solutions would be more targeted to ensure the overall safety and effective deployment of service robots. Here we have decided to explore and apply the FMEA model as an alternative hazard analysis approach. We adapted the FMEA approach to better relate failures to both the properties of the robot and the design of the building environment it operates in.

### Adapting current FMEA to robot-inclusive FMEA

In consideration of robot safety in our adaption of the FMEA method, robot-inclusive design principles were used as a categorisation tool and framework to analyse failures. The robot-inclusiveness is defined as the metrics for evaluating how much the design of the environment takes into account robot safety. Five robot-inclusive design principles, namely: safety, accessibility, activity, observability, and manipulability, were inspired by and derived from universal design methods^52,53^. Safety is the overarching principle which serves as a foundational base to the other four principles. Accessibility involves maximising the robots’ area coverage of its workspace, providing barrier-free access and connectivity for robots to travel for their tasks. Activity aims to provide for the efficient integration of workspaces between people, goods and robots. The Observability principle works towards improving the spatial environment for robot visibility and perception of its surroundings for navigation and carrying out tasks. Finally, Manipulability strives to enhance the robot’s ability, if any, to move or rearrange objects in the robot’s given environment with more precision and success using its end effectors. These principles would provide an advisory structure for improving spatial environments and work zones to allow for effective robotic deployment.

**Figure 2:**
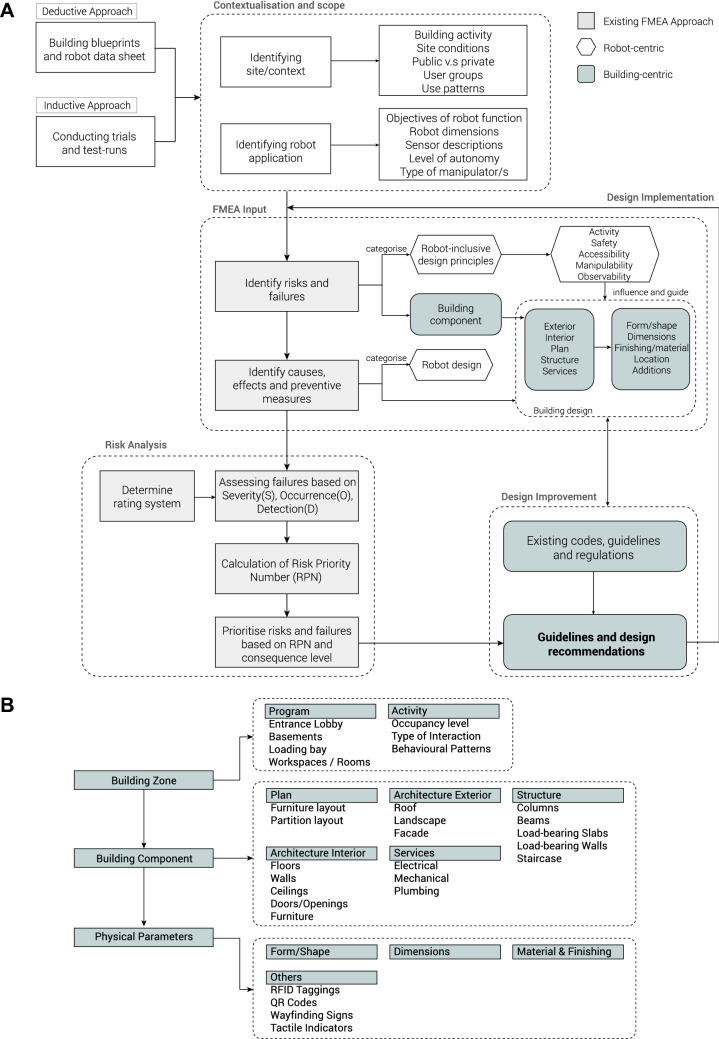
RIFMEA framework relating robot-centric and buiding-centric principles. **(A)** Workflow diagram of proposed RIFMEA method. **(B)** Building system diagram.

The proposed RIFMEA approach helps to provide an evaluative framework to assess the safety of the built environment in the application of robots by identifying and analysing safety hazards. Fig. 2A illustrates the workflow of the proposed RIFMEA approach, highlighting our additions and changes to the common FMEA method. Typically, the common FMEA approach for robot safety only considers the determination, classification and analysis of the failure modes found in the robot’s components. The RIFMEA method considers building component related hazards that are associated with the failure of the robot operation. Other than humans, both the safety of the robot and the building are considered in the risk and failure identification stage in the application of the RIFMEA method. Building components that resulted in robot operation failures are recorded and categorised under the five robot-inclusive design principles to provide clarity to the cause of the failure.

One of the crucial steps of applying RIFMEA method is breaking down the building system to individual building components. Having gained understanding of some existing frameworks to categorise the different systems and components that make up a building through referencing works from Bachman^54^, Rush^55^ and Brand^56^, we have proposed a categorisation structure for the RIFMEA framework. Fig. 2B illustrates this structure through a building system diagram.

The building is analysed in zones with their respective programs and activities. In each zone, the building components are broken down into five sub-categories as shown in Fig. 2B. These building components can be further subdivided into its various building elements. For example, the architecture interior building component can be further broken down into its constituent building elements such as walls, doors, furniture, floors and ceilings. Finally, the building components can be analysed by their physical parameters. The building system diagram shows how the different parts of the system interact with one another to gain better understanding of its correlation to the failures occurred.

#### Effects and causes

When evaluating the failures, the cause of the failure can be traced back to the components of the robot and the physical parameters of the building components. Robot components can be categorised into locomotion mechanism, body frame, sensors, and the manipulators or end-effectors. Similarly, the physical parameters of the building component can be categorised into different aspects such as its form or shape, dimensions, material or finishing. Understanding the cause of the failure can help to propose more targeted recommended actions to either reduce the severity and occurrence, or improve the ability in detecting the failure. For our scope, we are mainly interested in looking into causes of failures that relate directly to the building components and elements. When failures are tagged to building components, the physical parameters of the building components or the lack of, are often the cause of failures. Adjustment and alterations to these physical parameters serve as actions that can be taken to alleviate the failures.

After carrying out a thorough analysis listing of causes and effects, Risk Priority Numbers (RPNs) of each failure were calculated after allocating rating scales for severity, occurrence and detection. High priority failures that were detected and analysed based on the degree of mismatch of building components, its building elements and physical parameters to the safety of the robot’s operation. Following this, specific building design suggestions by considering changes that can be made to the physical parameters such as changes to the material and finishing or form are proposed to reduce or prevent such robot-related accidents. These design suggestions should take into consideration the existing building codes, guidelines and regulations which account for human safety and ergonomics.

#### Modified S,O,D rating system

Individual S,O,D rating systems were developed to make it applicable for the context of deploying service robots in an environment, examining the building components as the subject of interest. A clear establishment of the scales would help towards impartiality when assigning scores to the respective ratings^33^. RIFMEA also adapts its rating system from the military standard MIL-STD-1629A drafted by The United States Department of Defense^57^. FMEA was first applied by the U.S. Army, to which it provides a reliable foundation based on its application in complex military systems. Furthermore, its generic applicability allows it to be adapted and employed in multiple industries such as automotive, aeronautical, nuclear and electro technical^58^. Various FMEA studies on robot safety^59,60^ as well as robot-human interaction^61,62^ have also adopted the MIL-STD-1629A standard to conduct the FMEA. The adapted S, O and D rating system for RIFMEA can be seen in Table 2 below. All the three scales were modified to incorporate a 1-5 scale instead of the existing 1-4 scale for the provision of a neutral grade^63^.

**Table 2:** S,O,D rating scales for Robot-Inclusive FMEA. **(A)** Severity rating scale. **(B)** Occurrence rating scale. **(C)** Detection rating scale.

A				
Severity rating	Human	Robot	Environment	Additional remarks
1	No injury	Robot has to reorientate itself, robot endures moderate performance drop	No damage	Robot is able to complete its task
2	Minor injuries: burns, temporary scarring	Robot has to reorientate itself, robot endures high performance drop	Minor damage/scratches	Robot is able to complete its task
3	Non-incapacitating injuries	Robot continue operations, but operations become limited	Major damage to building component	Robot is able to complete its task
4	Incapicitating injuries	Robot has to cease operations and has decent damage	Severe damage to building component/Parts have to be rebuilt or replaced	Robot cannot carry out its task
5	Fatality and permanent serious injury	Robot has to cease operations and suffers serious damage	Severe damage to surrounding building components and building component does not comply with building regulations	Robot cannot carry out its task

B				
Occurrence rating	Probability score (x)	Occurrence		
1	x< 0.125	Very low		
2	0.125 < x < 0.375	Moderately low		
3	0.375 < x < 0.625	Average		
4	0.625 < x < 0.875	Above Average		
5	0.875 < x $\le$ 1.0	Very high		

C				
Detection rating	Human operation	Autonomous operation	Detectability	
1	Users will always detect and prevent the failure	Robot will always detect and prevent the failure	Very high	
2	Good likelihood that users will detect and prevent the failure	Good likelihood that robot will detect and prevent the failure	High	
3	Moderate likelihood that users will detect the failure	Moderate likelihood that robot will detect the failure	Moderate	
4	Low likelihood that users will detect the failure	Low likelihood that robot will detect the failure	Low	
5	Very low (or zero) likelihood that users will detect the failure	Very low likelihood that robot will detect the failure	Very low	

The modified Severity scale seen in Table 2A considers three different entities—robots, humans and objects in the robot’s working environment were considered when assessing the severity of failure modes. In this case, humans were given utmost priority, followed by robots and building components. This is because the design of the built environment is first considered for humans before robots. The five categories of severity for human injuries are developed based on existing works in machinery risk assessment^64^ and the area of workplace safety^65–67^.

The modified Occurrence scale in Table 2B considers the extent of likelihood a failure will occur given that the robot is run under similar environmental settings. The probability of the failure happening is given a score ranging between 0 to 1, and is calculated as a fraction of the number of times a particular failure occurs over the number of times the robot interacted with the building element of interest, as illustrated in the Eq. (1) below. This probability score will correspond to an evenly distributed Occurrence rating scale of 1 to 5 whereby a high probability score of more than 0.875 would be equivalent to a Occurrence rating of 5, while a very low probability score of below 0.125 will be equivalent to an Occurrence rating of 1.

1 $$\begin{aligned} \chi = \frac{\text {Number of times failure occurred}}{\text {Number of interactions with the building element}} \end{aligned}$$

The Detection rating accounts for the likelihood of detecting the failure before it can occur. The rating is dependent on the level of autonomy of the robot, whether it be teleoperated, semi-autonomous or fully autonomous. Semi-autonomous robots have to consider both the detectability by the human operator of the robot as well as the autonomous detection capabilities of the robot. The scoring is shown in Table 2C.

With the resultant RPN scores and corresponding location tag for all failure modes, hazard maps can be created for the robot to plan better robot task routes to avoid hazardous locations. Actions can also be taken to restructure a particular location if the RPN score is high to make it more robot-friendly. Priority should thus be given to address failures with higher RPN values which correspond to those of higher risk.

### Level of spatial adaptability

For spaces shared by both human and users, making alterations or changes to the environment to help improve robot safety would inevitably affect how humans use or experience the space. While robots are proposed as new stakeholders in the built environment, priority should still be given to human safety and humans’ usage of the space, where applicable. It should support the common forms of HRI, namely coexistence, cooperation or collaboration^26,68,69^. Alterations to a space to make it robot-inclusive should not render it impractical or unusable for human users.

When it comes to restructuring a space, we acknowledge that the different building components have varying levels of spatial adaptability. Adaptability in buildings refers to the building’s capacity to accommodate change^70^. For example, altering the design or relocating the columns (Structure) would be much more cumbersome than simply changing the furniture layout (Plan) of a space. At the same time, there can be different solutions of varying levels of adaptability to address a single problem, albeit with varying effectiveness. For instance, to rectify the hazard of having disorganised, loose cables on the floor, one could consider rewiring the electrical cables into the flooring or adopting cable management means such as installing cable trunking to reorganise the wires. The former would likely involve more cost and effort than the latter, but may be more effective in eliminating the hazard altogether. Understanding the ease of adaptability would thus affect the planning towards achieving robot-inclusive spaces.

## Methods

### RIFMEA worksheet

Based on the work^33^, the FMEA worksheet was adapted to develop our own RIFMEA worksheet. A sample of a completed worksheet can be found in the supplementary materials. Observations of failure modes were recorded on the proposed RIFMEA worksheet, along with the intended actions the robot was meant to execute, the causes of failures and their effects. Some of the key components of the robot such as the locomotion mechanism and sensors installed are included in the worksheet. The different failures are categorised accordingly into their building components and the respective elements, depending on what the robot was interacting with at the time of failure. Moreover, the corresponding robot-inclusive design principle that applies to the failure is noted down. This gives a better clarity in what way the building component has failed in terms of the robot-inclusive principles, examples being issues limiting the robot’s access (Accessibility), or the robot’s perception of the space (Observability). This would help to better relate the failures to their causes and in turn provide insights on design improvements to be made to the environment or the future iterations of the robot, or both. The individual failures were analysed to determine their S,O,D scores and the eventual RPN. Finally, recommended actions dependent on the failure types would also be noted down.

As shown in the workflow diagram in Fig. 2, we identify two main approaches in carrying out the RIFMEA framework: inductive and deductive. The inductive approach refers to analysing failure modes and risks through carrying out actual test studies by deploying service robots physically in an environment, observing and recording all the various faults during the test runs. The deductive approach, as the name suggests, deduces and identifies potential threats based on the analysis of the building drawings or blueprints as well as that of the robot, keeping in mind the robot’s mode of locomotion, physical dimensions, degree of autonomy, sensors equipped and manipulators if any. A familiar understanding of the properties of both the building and robots of interest would greatly help in the deduction of potential hazards and failures.

Regardless of the approach adopted, it is important to have a comprehensive understanding of target operational requirements of the robot, be it maximising area coverage, or travelling smoothly across designated waypoints in the case of our case study. This would better help deduce the potential failures that hamper the robots’ productivity or efficiency based on the operational requirements.

While the inductive approach can only be applied and tested on existing robots and buildings, the deductive approach can be implemented to study robot applications that may not be yet realised, be it either the development of the robot or the construction of the building of interest. Prior findings from robot experiments in existing buildings under the inductive approach would help to deduce and assign those values during the building design phase. As this paper takes a first attempt at applying the RIFMEA, we would only be looking at the inductive approach. With subsequent studies using the inductive approach, we can then attain a better understanding and knowledge of robot safety in buildings to carry out the deductive approach on future developments, identifying hazards without carrying out actual test runs, economising on both time and resources.

### Contextualisation and scope

#### The robot: Double 3

**Figure 3:**
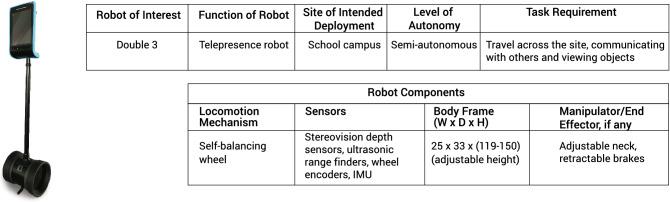
Double 3 robot description and components.

As an application of this proposed RIFMEA framework, the commercial Double 3 telepresence robot was deployed in various settings within the Singapore University of Technology and Design (SUTD) campus. Telepresence robots allows users from another location to participate in activities through the robot remotely. The Double 3 robot is a commercially available mobile telepresence robot that is utilised in many research studies^71–73^. It is a reliable, simple-to-use technology that can be easily implemented in various settings. According to their website, this commercial product has also been deployed in multiple universities in the United States (US) as well as businesses and healthcare environments. The form factor of the Double 3 robot is shown in Fig. 3.

The Double 3 robot has two, 13-megapixel pan-tilt-zoom cameras (wide-angle and super zoom), 6 beamforming microphones and a speaker. It has an adjustable neck with a maximum head height of about 150 cm, weighs about 7.3 kg and runs on two wheels on a self-balancing base. Attached to its head are five ultrasonic distance sensors and a pair of Intel RealSense D430 depth sensors on its head to sense obstacles on the floor and ahead of it. The depth sensors also help to generate a 1280 $\times$ 720 depth data with a range of between 0.2 and 10 meters away^74^. With the depth data, an augmented reality screen showing what the robot senses and sees is provided for teleoperators and an overlay of a grid of dots denote the areas accessible for the robot. To navigate, the teleoperator designates a goal point on the accessible areas shown on the interface screen in real time as the teleoperator views the scene through the cameras on the robot. The robot itself then performs obstacle avoidance and path planning to navigate towards the given goal. Alternatively, the robot operator can also move the Double robot using the arrow keys on the computer keyboard.

Although the Double 3 robot was used only as an application for the proposed framework, the methodology of the framework shown in Fig. 2 can be extended to assess the safety of other robotic deployments in other environments as well. At the current stage, it is difficult or almost impossible to generalise the safety of all robots using the deployment of a single robot as there exists a wide range of robots carrying different sets of sensors with varying capabilities and physical dimensions. In other words, what is safe for one robot may not be necessarily so for another. Yet, it would also be impractical to perform assessments for all permutation of robots to test their safety for each of them. Instead, the outcomes of the study would be valid for other robots that use similar sensing technologies as the Double 3 robot.

#### Test locations within university campus

**Figure 4:**
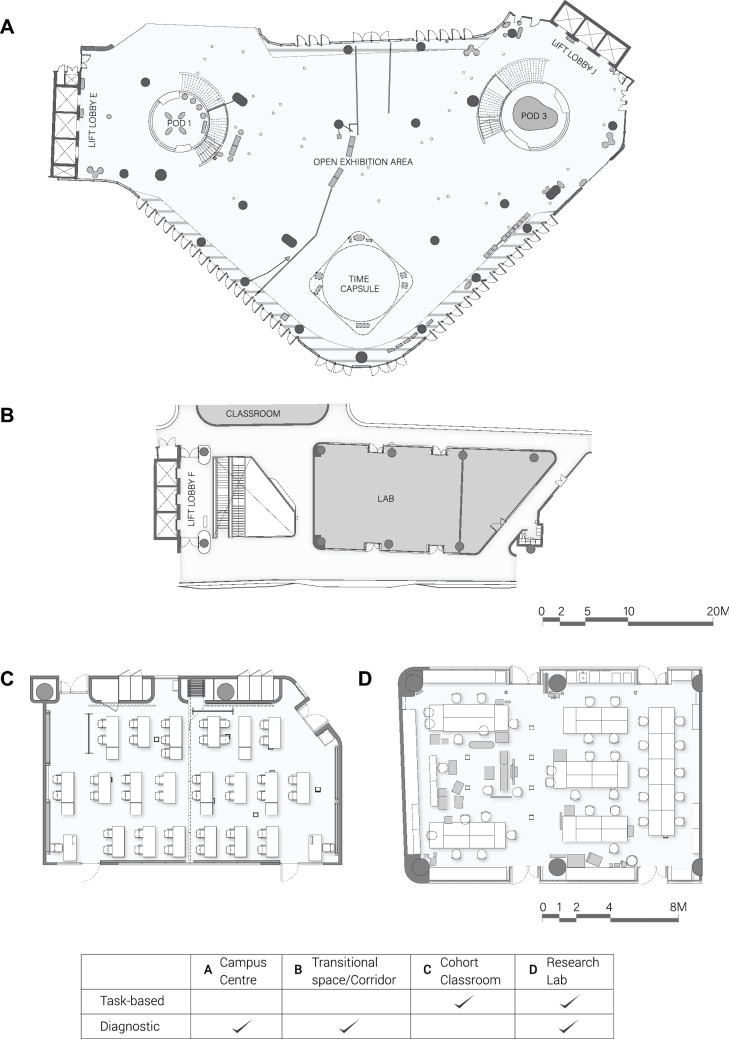
Plans of test sites and test type breakdown. **(A)** Campus Centre. **(B)** Transitional space. **(C)** Cohort Classroom. **(D)** Research lab.

Several test locations were identified within our campus site: a classroom (also known as a Cohort Classroom), a research lab, the main plaza (also known as the university’s Campus Centre), and a transitional space which included common corridors and lobbies. These locations were chosen for being common places where the telepresence robots could be used and deployed for different use scenarios, such as to attend or conduct a class, view objects of interest, or engage in team discussions and consultations. The floor plans for these areas are seen in Fig. 4.

The classroom is the common learning environment for students and teachers. We identified that lessons are usually conducted in two general ways: one whereby lessons are conducted in a more top-down approach where a teacher stands in front of the class and conducts a lesson, and the other in which learning takes on a project-based approach with teachers or facilitators moving from one student or student group to another, engaging in consultation sessions while making references to physical prototypes or models within the classroom. In addition to common walkways or corridors, the campus centre is also identified as a key test location, being an extension of the transitional space, capable of accommodating larger groups of users, gatherings and holding of events such as exhibitions or fairs. Finally, the research lab provides opportunities in the use of the robot in a non-optimised arrangement of interior fittings, where the environment is slightly more confined and even cluttered at times. Prototypes are being worked on through different stages of design within the lab, allowing for the use of robots to allow users to engage in discussions or monitor processes and workflows remotely. These locations would help provide a variety of spatial environments within the campus to test the application of the RIFMEA approach in evaluating building safety for robots.

### Testing procedure

Next, a procedure was developed on how the test runs would be conducted in each location. In the paper^27^, Dogramadzi et al. defined the concept of mission tasks and non-mission tasks. Mission tasks referred to the jobs that would contribute to fulfilling its intended function. Observing hazards relating to mission tasks would allow one to “handle the expected interactions of the robot with its environment”. On the other hand, non-mission tasks were defined as tasks apart from mission tasks that would be necessary for the robot to remain ready for operation and deployment whenever the robot is not necessarily performing a specific task. Consideration of hazards relating to non-mission tasks would allow one to “handle the unexpected interactions”. Adapting this thought process into our paper, the testing procedures were drafted out and conducted to help us consider both expected interactions and unexpected interactions by running the robots diagnostically as well as in a task-based setting.

In conducting a diagnostic test, the robot was made to navigate comprehensively throughout the site, covering all parts of the site as much as possible by moving along the inner perimeter and outwards in a spiral pattern towards towards the outer perimeter of the site, or from one end of the room to the other in a zigzag pattern. Observations of failures relating to the five robot-inclusive design principles were recorded as the robot moved around the site. In addition, the intended and actual paths taken by the robot were also recorded.

To consider hazards relating to mission tasks, we adopted the use of Hierarchical Task Analysis (HTA) approach to consider the various actions the robot would be required to carry out at each stage to achieve its main objectives. These interaction-related tasks were identified based on typical uses of the telepresence robot, which are also defined for our tests. For example, in the scenario where the telepresence robot is used to hold a discussion between two parties, the main sequence of tasks involves: a)logging into the robot, b) moving to its required location, c) viewing the object of interest and communicating with the other party, and d) returning to its docking station. Each of these tasks can be further broken into sub-tasks such as identifying traversible areas, carry out path planning and detecting and avoiding obstacles to move to its target location. Carrying out this step-by-step analysis of the task sequence will help to identify and spot failures when running the task-based tests. In the various tests conducted, the starting conditions of the robots were noted. They were either pre-positioned in place for immediate operation in its desired location, or the robots began their operation starting from their respective docking stations at designated locations. The locations of the docking station were also noted. Similarly, the spatial conditions of terminal locations were also studied; in most scenarios, the robot was tasked to return back to the docking station through its auto-docking mechanism. In this way, the start and end points of the robots are thus clearly defined to ensure that the whole deployment process and path are taken into account.

As transitional spaces such as the corridor and the Campus Centre are more open-ended in their program whereby the use and route of the telepresence robot is often undefined, we have decided to run diagnostic tests in these areas. This would provide a more comprehensive overview of possible hazards the robot could encounter in these environments, especially from users that are using the robot to navigate in an unknown space with the telepresence robot for the first time.

For the classroom and research lab, the telepresence robot was utilised in an activity-specific manner within a more confined environment. Here, we ran a task-based run with the robot. For a task-based run, the robot was made to execute a list of specific tasks. The paths which the robot took were not pre-planned but left completely to the teleoperator’s decision to direct the robot within the various spatial settings.

### Documentation of the applied RIFMEA

Besides the RIFMEA worksheet, a plan drawing of the test site as shown in Fig. 4 is required to denote the location of hazards that occur during the test. In this manner, the actual path taken by the robot is also recorded using the plan. As an illustration of the procedure and documentation process of the RIFMEA approach, the method and findings of test runs conducted at the research lab is presented in this section.

**Figure 5:**
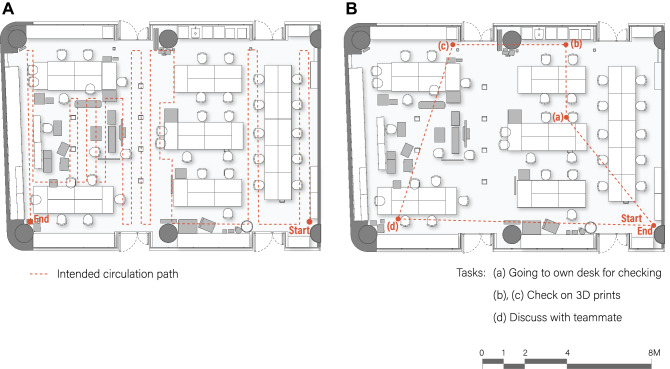
Intended circulation routes for the research lab. **(A)** Vertical diagnostic route. **(B)** Task-based route.

The intended path of the robots were first determined prior to conducting the test. Both diagnostic and task-based test runs were conducted in the research lab. For the diagnostic test, due to the rectilinear layout of the lab, the test was split into two parts: vertical-diagnostic and horizontal-diagnostic. The robot would start at one corner of the room before covering the majority of the space in a zig-zag manner. In the task-based test, a series of tasks which the robot was to perform in order was determined alongside its start and end point. The user was free to decide on the route to take to perform the set of tasks. As an illustration, the intended path for the vertical-diagnostic test, and the sequence of required tasks for the task-based test are shown in Fig. 5.

**Figure 6:**
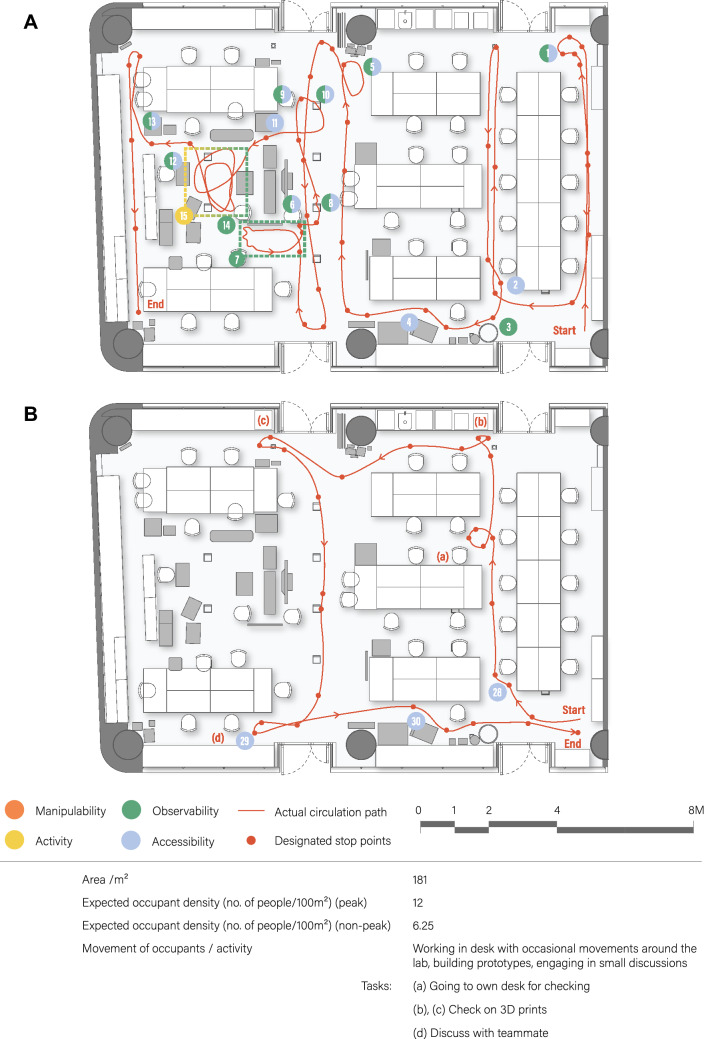
Result documentation for the research lab. **(A)** Vertical diagnostic test. **(B)** Task-based test.

Next, the actual path taken by the robot as well as the hazards that occurred during the test was recorded spatially on the plan. The results of the vertical-diagnostic test and the task-based test are illustrated in Fig. 6A, B. The failures are numbered and tagged in relation to the position of the building element of interest in the context of the lab. Each failure and the corresponding number is recorded onto the RIFMEA worksheet, together with the analysis of the failures’ cause, effect, S,O,D ratings and the recommended action.

## Research limitations and future work

An assumption made during the test runs was that participants operating the robots already had some level of acclimation with the controls and interface of the telepresence robots prior to the test. Moreover, participants were briefed beforehand regarding the capabilities and functionalities of the robots. For this research, our interest was constrained only to limitations imposed by the design of the environment as opposed to limitations caused by the robot’s design. Our findings did not encompass users that did not have experience with the telepresence systems to minimise errors caused by the teleoperator’s lack of understanding of the robot or its interface. The study was conducted in typical use environments during the daytime. Future work would consider differing lighting conditions and how it might affect the performance of the robot’s sensors.

Having different stakeholders performing the RIFMEA would be ideal to generate a more comprehensive RIFMEA record, since each stakeholder group would have different perspectives and requirements for the robot deployment and operation to suit their respective needs^33^. This would allow the RIFMEA to be comprehensive and satisfy conditions set by each stakeholder group as much as possible. As an initial study, a limitation of the research work was that the RIFMEA tests were conducted solely by researchers and without other domain experts such as facility or operation management staff, which is a recommended practice in industry for conducting FMEA. For subsequent tests, more expertise from various relevant fields should be called to observe and analyse the failures for comprehensive studies.

Another limitation of this study is that it did not implement the use of simulations. Physics-based simulations allow for identifying hazards that can be easily picked up by learned or pre-programmed models. Employing the use of simulations could reduce the time and manpower needed, especially when compared to conducting actual tests over large areas that have very similar spatial conditions. It could also act as a preliminary hazard identification method if it is impossible to carry out actual tests such as in early design phases of a construction. The use of simulations might also be more effective and practical when implementing simultaneous deployment of multiple robots where the robots are dynamically interacting with one another and with the environment^75^.

As a first attempt of the RIFMEA application, the method was utilised only over a few locations within a university campus with one type of service robot—a semi-autonomous telepresence robot. By conducting more test runs conducted across more spaces using different types of service robots, we would then be able to further develop a framework to evaluate and rate the building’s safety for service robots in a more holistic and objective manner. A larger data set would provide more opportunities and comparison across different spaces to draw any insights, new findings or more concrete conclusions, such as when comparing the average RPN values in different locations. Another potential area of study is to determine there might be any correlations between the building program or spatial quality and the safety for robots. This includes the occupancy level, programmatic function, user demographics and other factors. In addition to robot safety, further studies on robot performance metrics such as area coverage or time taken can also be conducted to draw relationships between spatial conditions to robot efficiency.

Moreover, the RIFMEA method is to be applied with other types of service robots; cleaning robots, inspection robots and other forms of maintenance and mobile service robots. The process of defining the site context and robot application, categorising the failures into the corresponding building components, and analysing causes with respect to both the robot and building, are extensible and applicable to assessing other robot deployments. These aspects of the methodology are also the main contributions of this research. Each robot varies in terms of its mechanisms and software, which include but are not limited to, its installed sensors, size, end effectors and locomotion mode. As such, how each robot perceives and interacts with the same given environment may differ. The hazard types as well as their corresponding Severity, Occurrence and Detection rating would likely also differ across each type of robot. With knowledge of the average RPN score across different built environments, the robot-inclusiveness of one environment for the deployment of service robots can then be compared to that of another.

Developing a comprehensive set of design guidelines and spatial improvements would be a next step to improve the robot inclusivity of buildings. While the paper presents some recommended actions to take in response to the various failure modes discovered, they are still quite generic and tend to be prescriptive instead of being a tool that would be useful for building designers to adopt. A more robust set of guidelines corresponding to the five robot-inclusive design principles should be developed to help improve the safety of robotic deployments in future buildings. This also includes the design of robot-inclusive furniture to help increase the accessibility, observability and overall safety of the robot.

## Conclusion

This paper has laid out the integration of robot-inclusive principles for generating an adapted robot-inclusive FMEA (RIFMEA) framework to assess building hazards for the deployment of service robots. Here, the robots are seen as a new stakeholder with regards to robot safety. This framework would aid the documentation and analysis of hazards for the service robots to be deployed, paying special attention to the different components of a buildings. Recommended actions can be taken based on the list of hazards, to prevent or reduce the impact of damage done to the robots during their operation. This would be a step towards improving the safety of the built environment for robots and allow for greater efficiency for the application of robots in buildings. The proposed RIFMEA is applied and tested using a commercially available Double 3 telepresence robot within various settings within a university campus. From the results of the RIFMEA performed with the Double 3 robot at the various areas, the failures were analysed and discussed upon, including some outlier cases.

## Supplementary Information

Supplementary Information.
