# Supplementary material for: Application of an adapted FMEA framework for robot-inclusivity of built environments
Source: Sci Rep. 2022 Mar 1;12:3408. doi: 10.1038/s41598-022-06902-4 (PMC8888750; doi:10.1038/s41598-022-06902-4)
Supplement: Supplementary file 1 — Supplementary Information. [file 41598_2022_6902_MOESM1_ESM.pdf]

Application of an Adapted FMEA Framework for Robot-Inclusivity of Built Environments

Y. J. Ng[1], Matthew S. K. Yeo[1], Q. B. Ng[1], Michael Budig[2], M. A. Viraj J. Muthugala[1], S. M. Bhagya P. Samarakoon[1], R. E. Mohan[1]

[1]Singapore University of Technology and Design, Engineering and Product Development, Singapore, 487372
[2]Singapore University of Technology and Design, Architecture and Sustainable Design, Singapore, 487372

Supplementary material

Contents

Annex S1. Intended routes of test runs and corresponding result path taken
Annex S2. Completed RIFMEA worksheet consolidating results from all tests

Annex S1. Intended routes of test runs and corresponding result path taken

Vertical Diagnostic

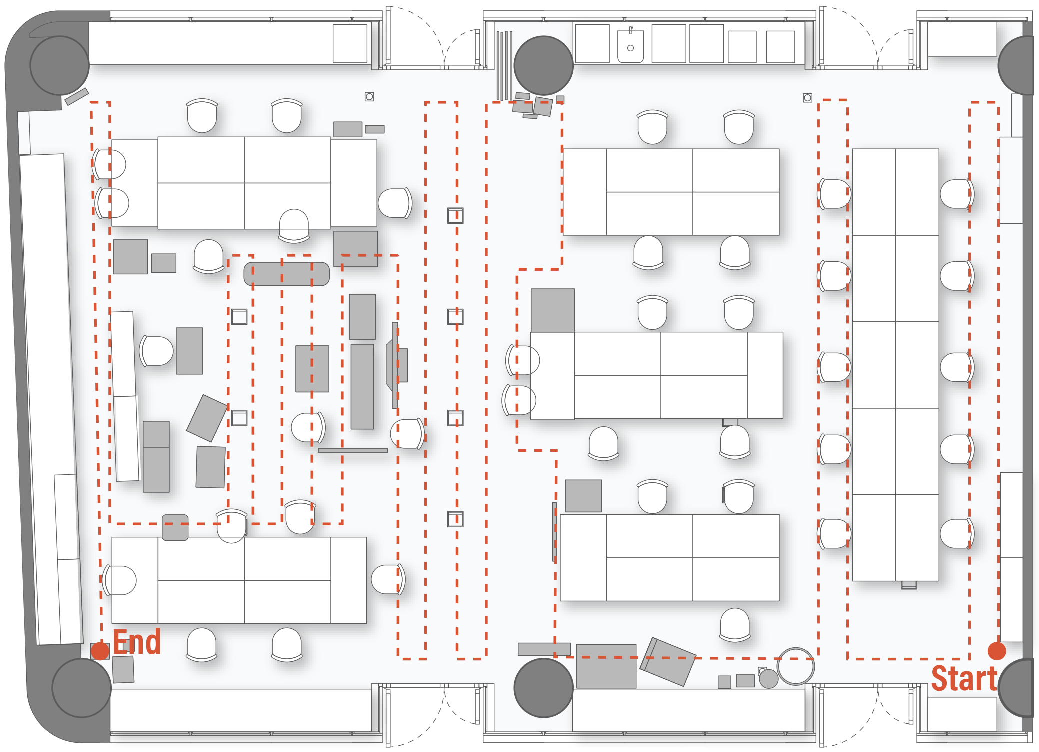

Horizontal Diagnostic

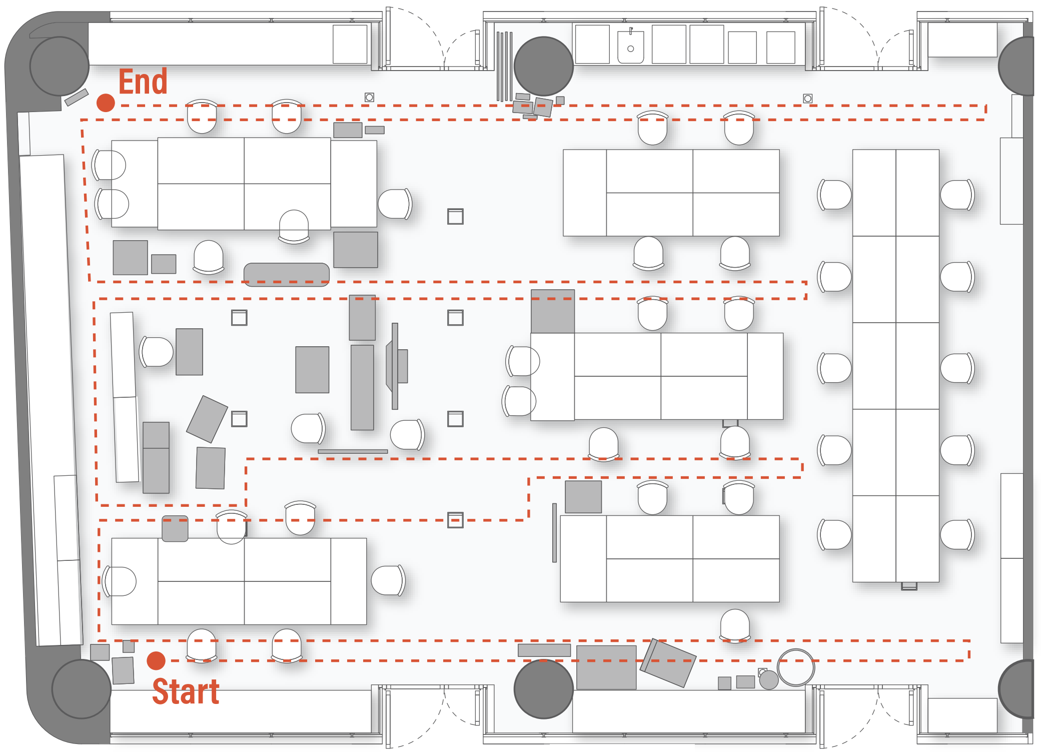

Intended circulation path

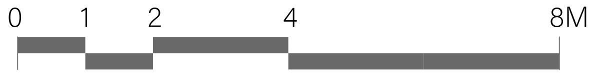

Research Lab

Area /m^2	181
Expected occupant density (no. of people/100m^2) (peak)	12
Expected occupant density (no. of people/100m^2) (non-peak)	6.25
Tasks:	(a) Going to own desk for checking
	(b), (c) Check on 3D prints
	(d) Discuss with teammate

Vertical Diagnostic

Horizontal Diagnostic

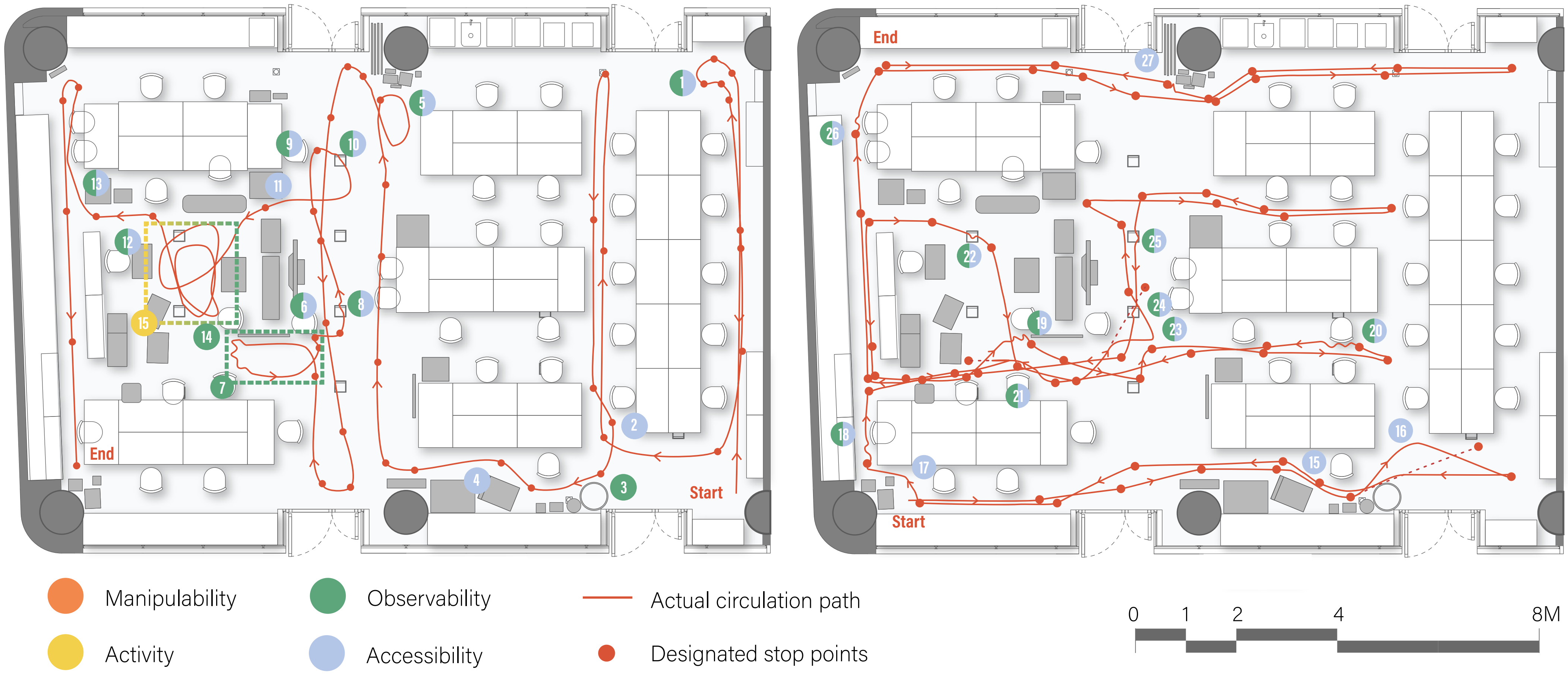

Area /m^2	181	Research Lab
Expected occupant density (no. of people/100m^2) (peak)	12	
Expected occupant density (no. of people/100m^2) (non-peak)	6.25	
Movement of occupants / activity	Working in desk with occasional movements around the lab, building prototypes, engaging in small discussions	

Task-based

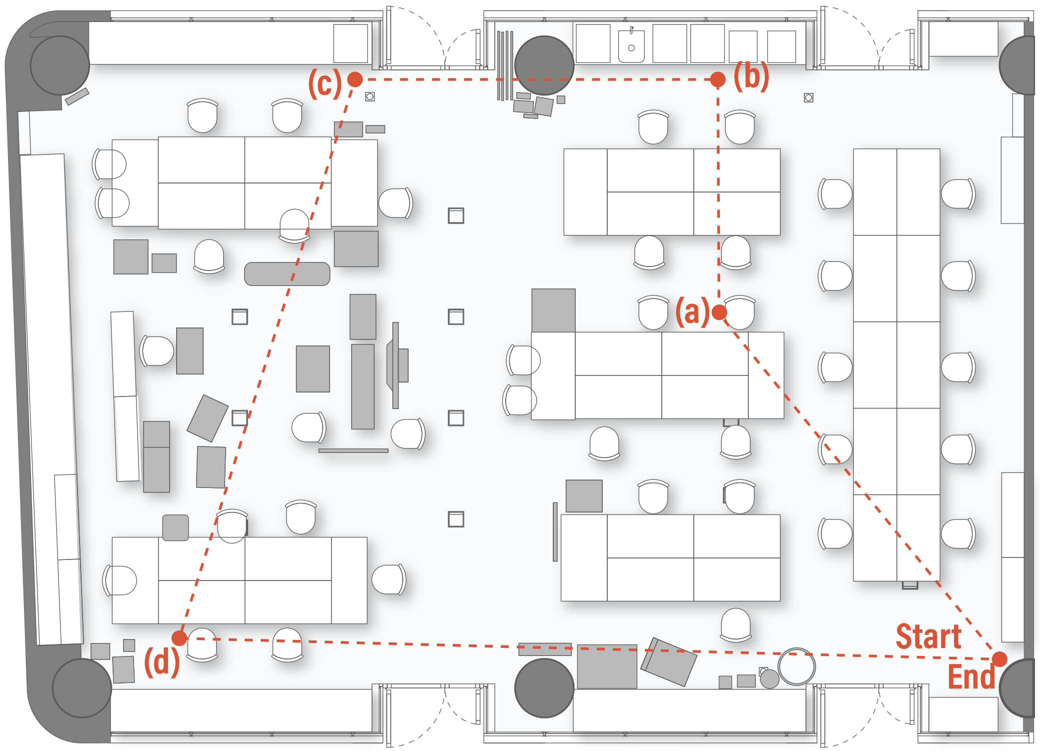

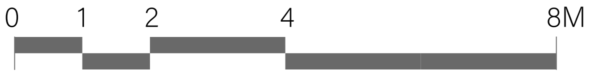

Research Lab

Area /m^2	181
Expected occupant density (no. of people/100m^2) (peak)	12
Expected occupant density (no. of people/100m^2) (non-peak)	6.25

Tasks:
(a) Going to own desk for checking
(b), (c) Check on 3D prints
(d) Discuss with teammate

Task-based

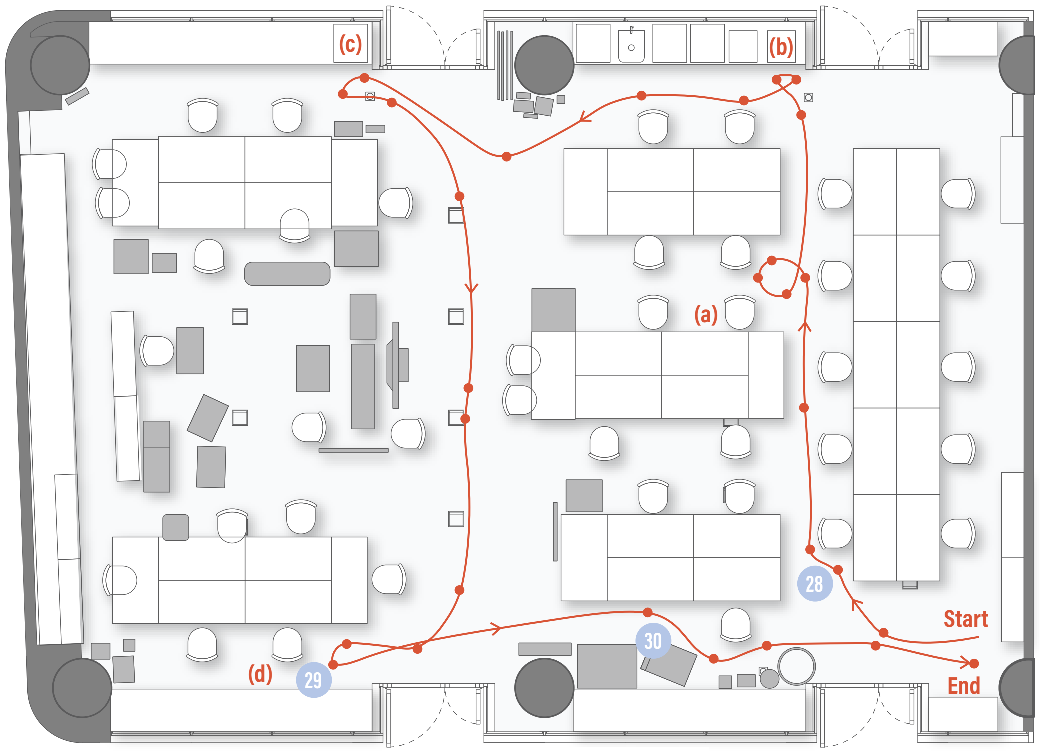

Manipulability

Activity

Observability

Accessibility

Actual circulation path

Designated stop points

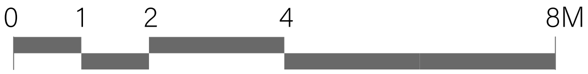

Area /m^2 181

Expected occupant density (no. of people/100m^2) (peak) 12

Expected occupant density (no. of people/100m^2) (non-peak) 6.25

Research Lab

Tasks:

(a) Going to own desk for checking

(b), (c) Check on 3D prints

(d) Discuss with teammate

Task-based

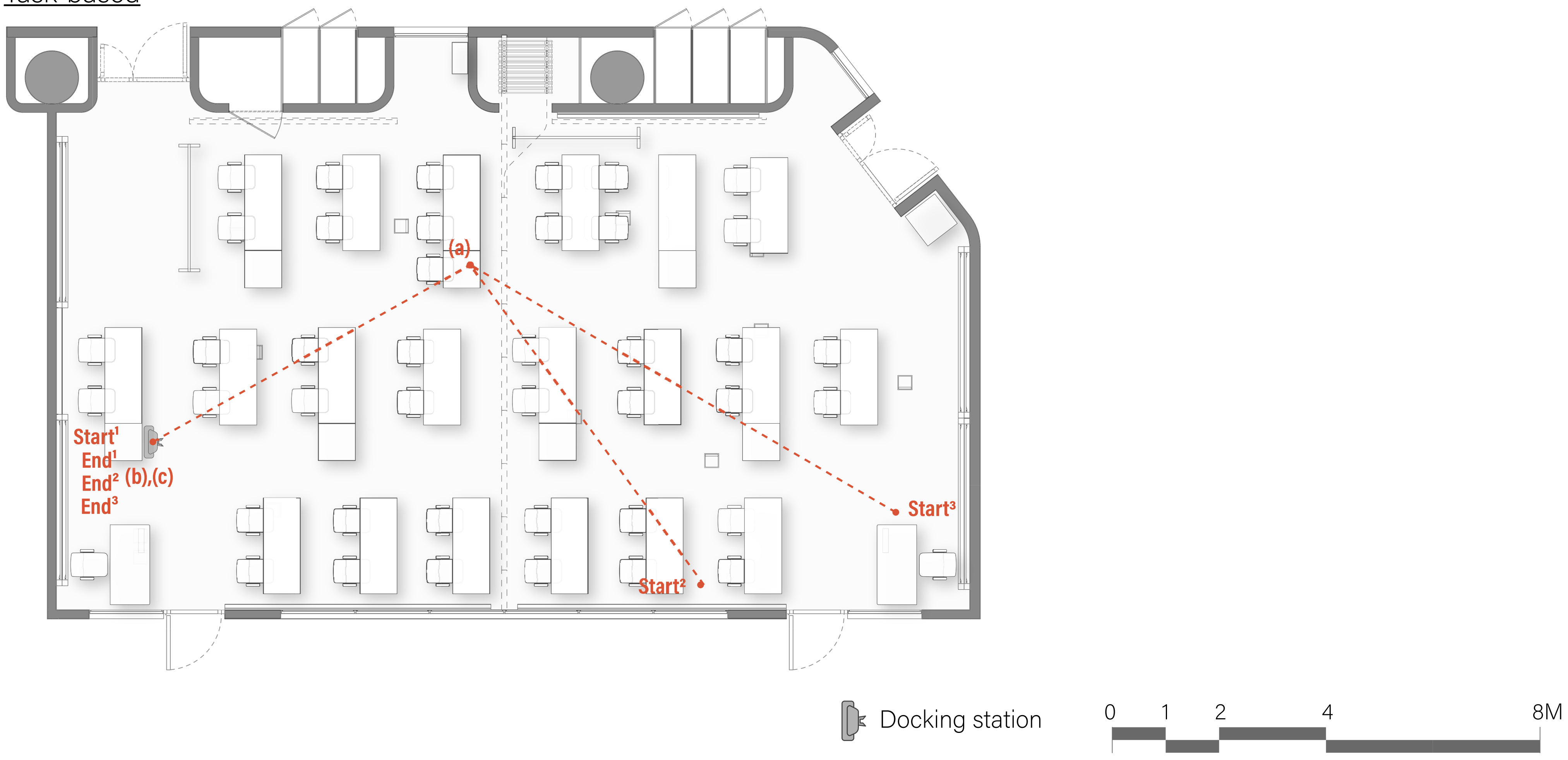

Area /m^2	164	Cohort Classroom
Expected occupant density (no. of people/100m^2) (peak)	18.3	
Expected occupant density (no. of people/100m^2) (non-peak)	6.1	
Tasks	(a) Go to group for discussion	* The position of the robot was moved after each user, hence the different starting points
	(b) Return to docking	
	(c) Switch user, repeat (a) - (c)	

Task-based

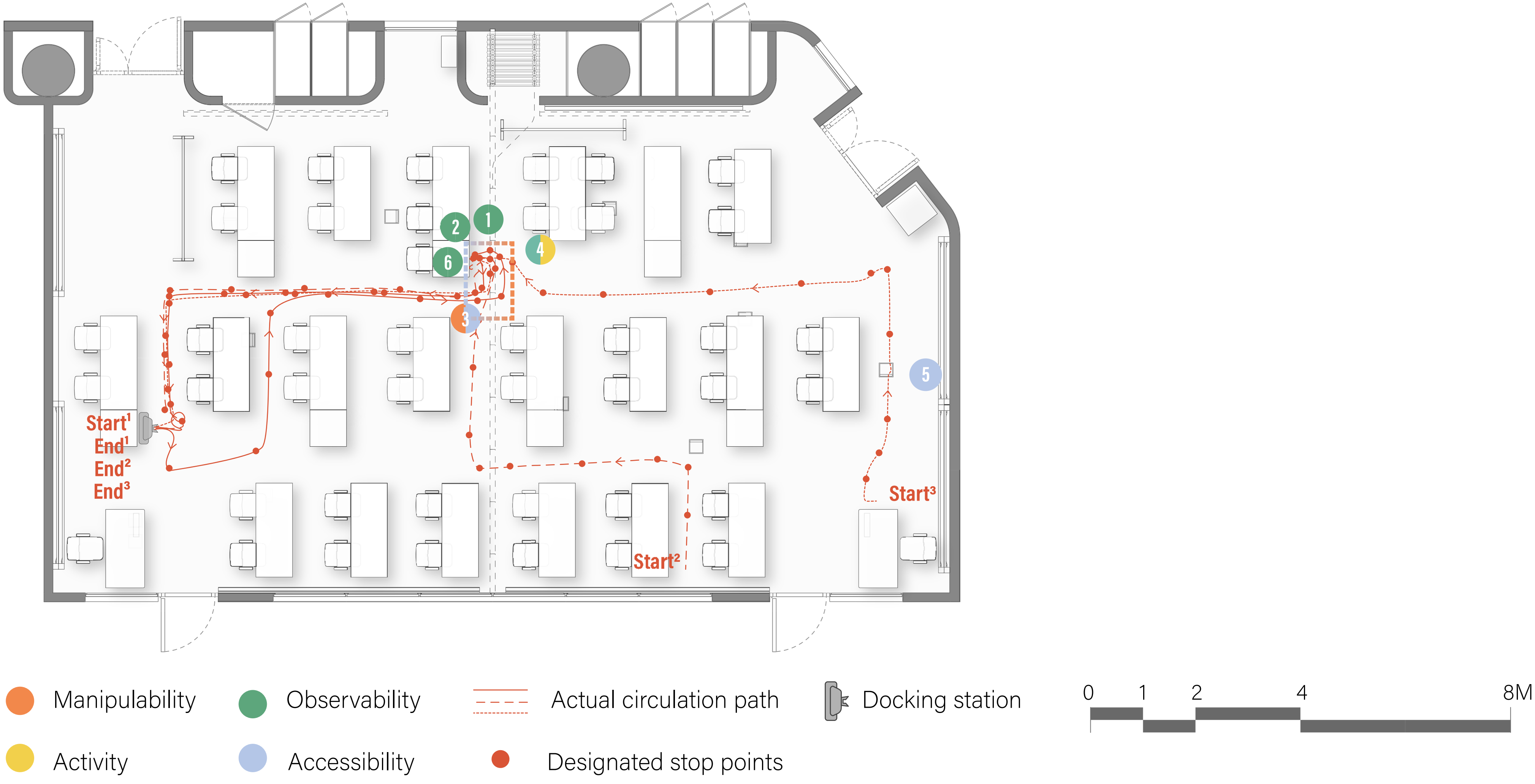

Area /m^2	164
Expected occupant density (no. of people/100m^2) (peak)	18.3
Expected occupant density (no. of people/100m^2) (non-peak)	6.1
Tasks	(a) Go to group for discussion<br>(b) Return to docking<br>(c) Switch user, repeat (a) - (c)

Cohort Classroom

* The position of the robot was moved after each user, hence the different starting points

Diagnostic

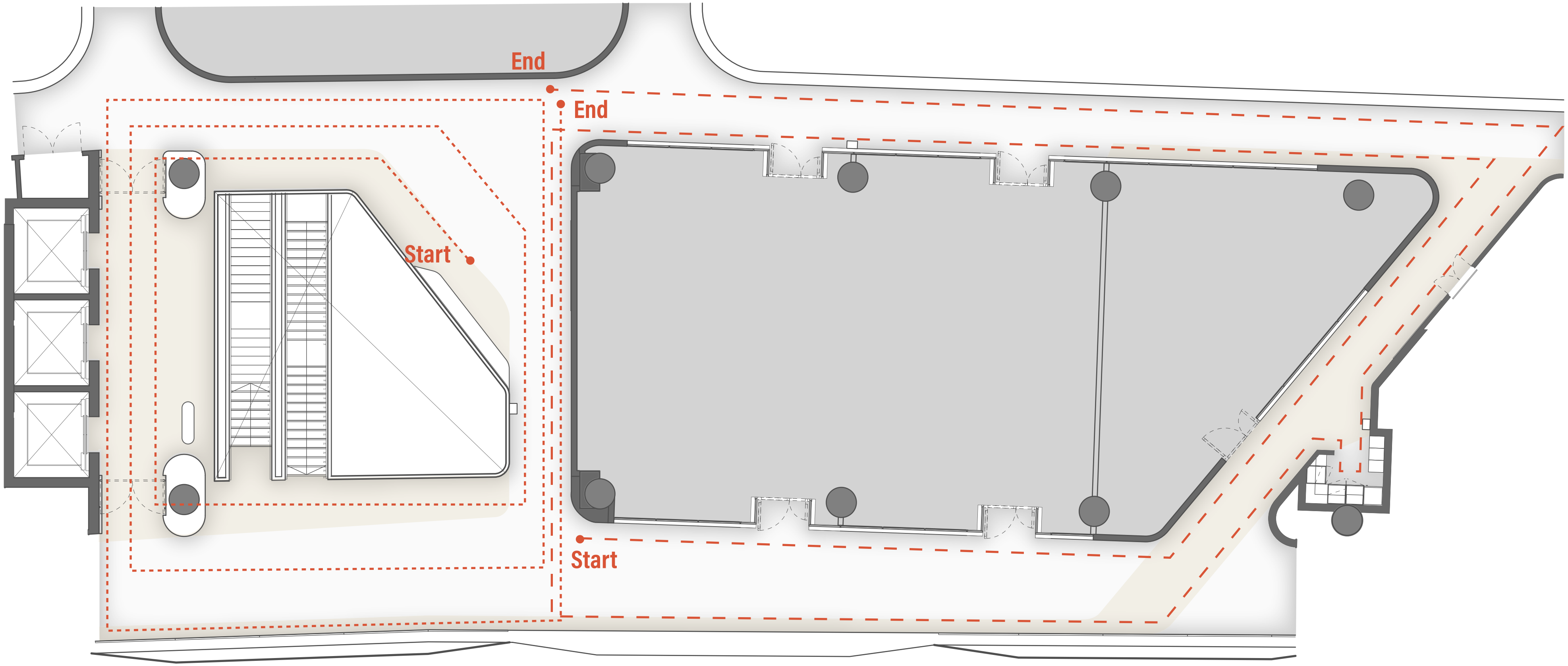

rough granite finish

smooth granite finish

Intended circulation path

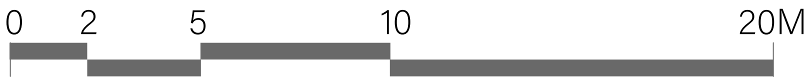

Area /m^2	421
Movement of occupants / activity	transient space for circulation and informal conversations

Transitional space

Diagnostic

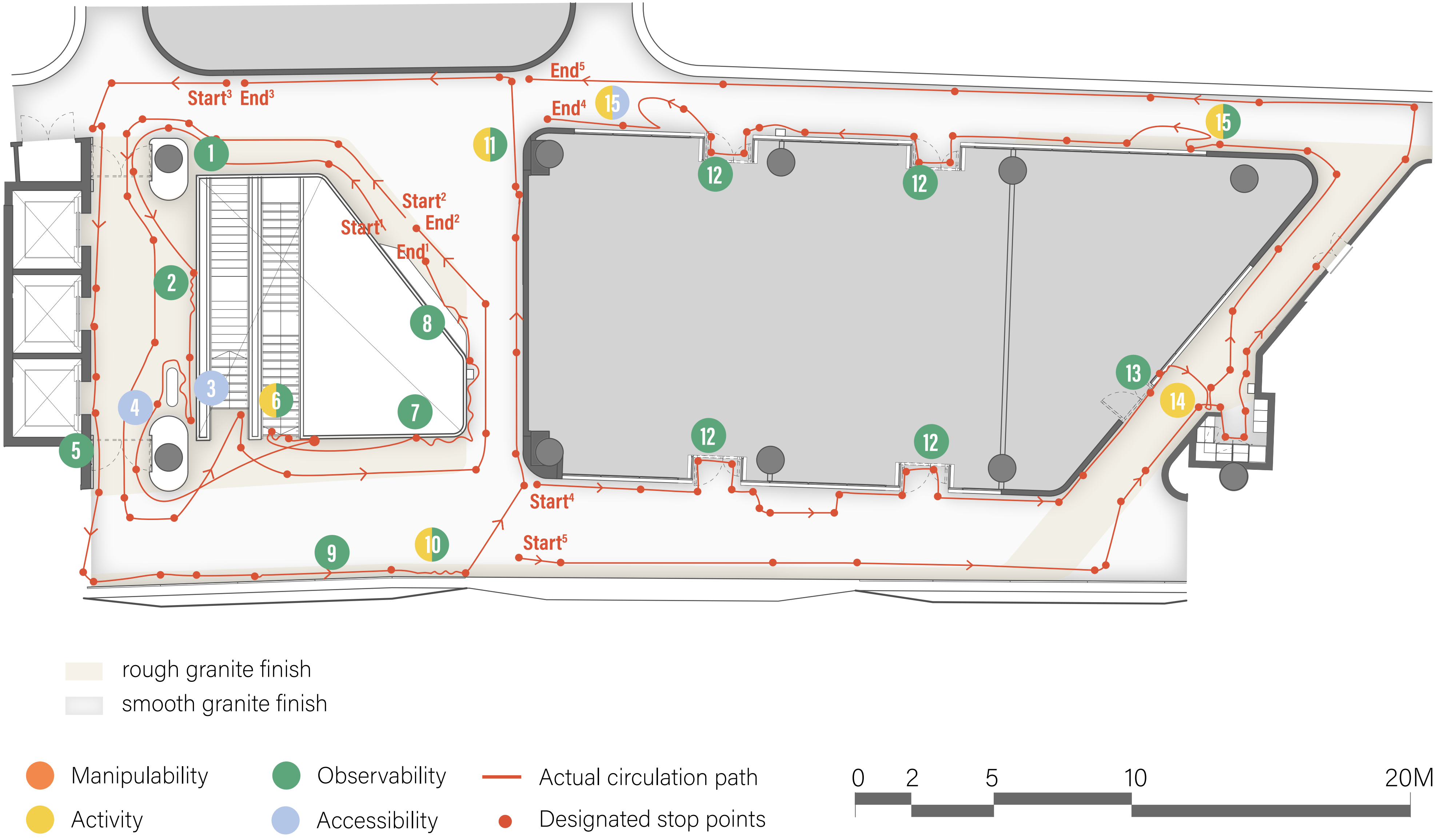

Area /m²	421
Movement of occupants / activity	transient space for circulation and informal conversations

Transitional space

Diagnostic

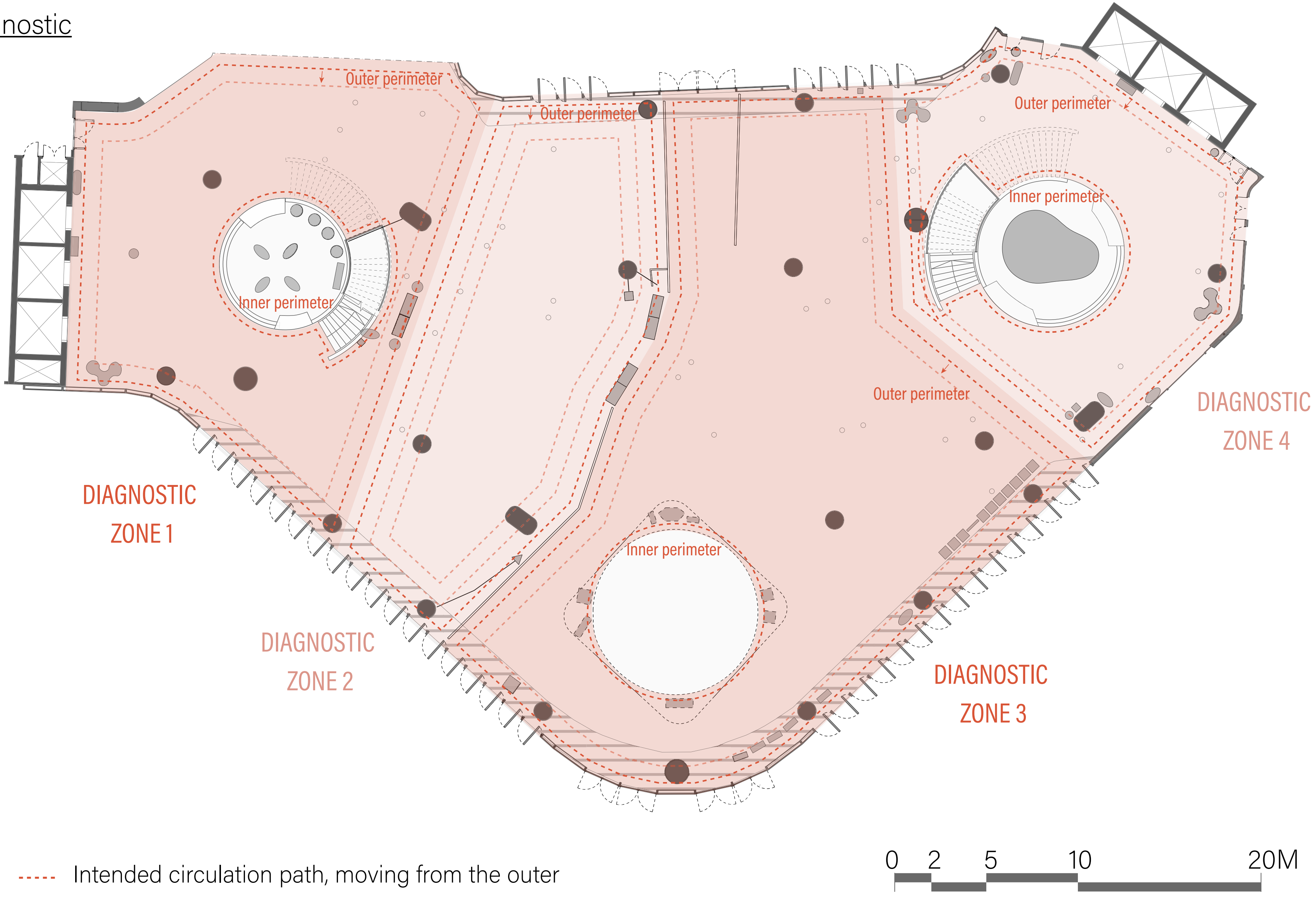

----- Intended circulation path, moving from the outer perimeter to inner perimeter in a spiral manner

---

Area /m^2	1766
Movement of occupants / activity	transient space for circulation and informal conversations, events, presentations, exhibition

Campus centre

Diagnostic

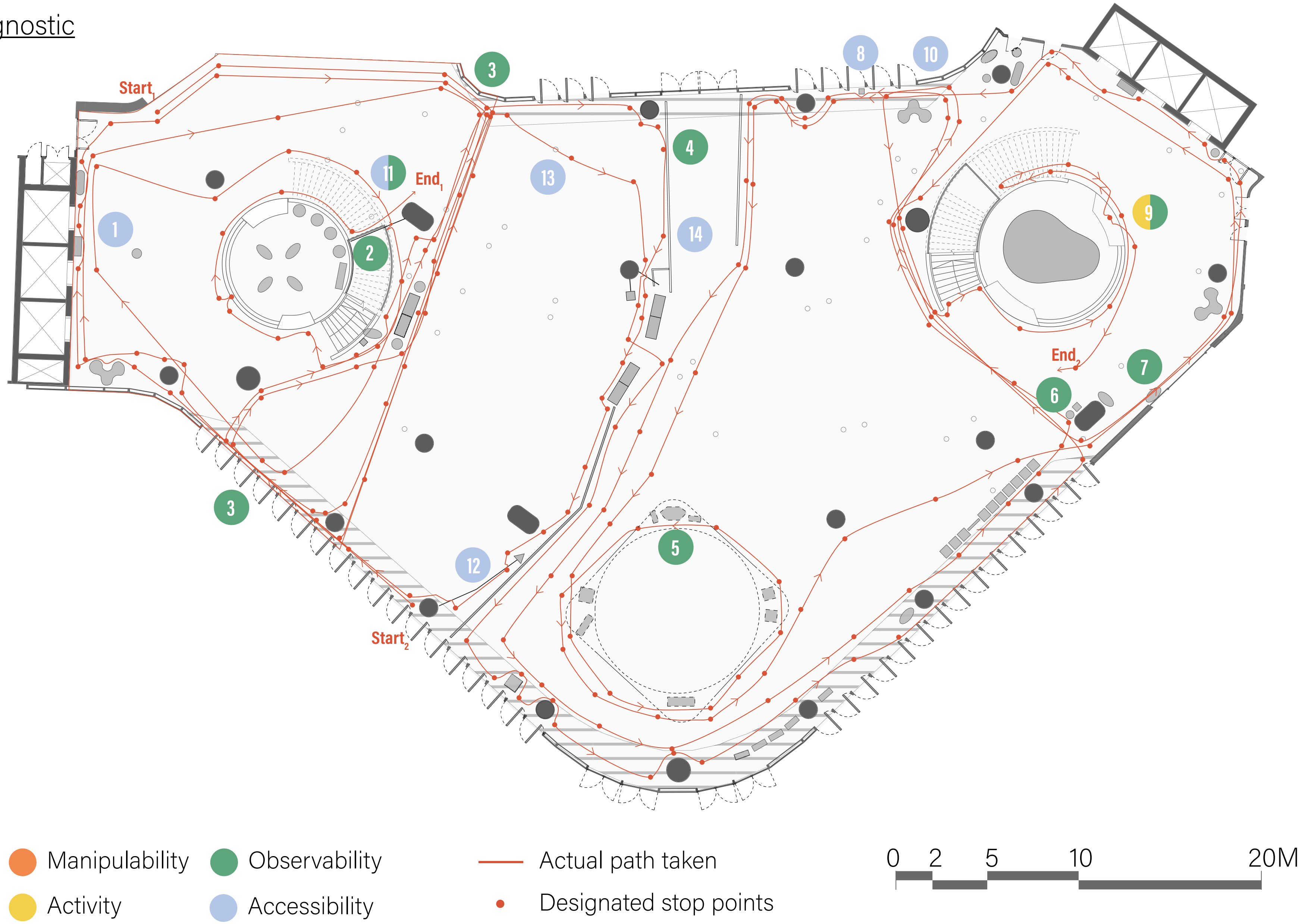

Area /m²	1766
Movement of occupants / activity	transient space for circulation and informal conversations, events, presentations, exhibition

Campus centre

Annex S2. Completed RIFMEA worksheet consolidating results from all tests

RIFMEA Study on SUTD Campus with Double 3 Robot

Robot of interest	Function of robot	Locomotion mode	Site of Intended Deployment	Level of Autonomy	Requirement	Sensors	Size (WxDxH)/mm, Weight/kg	Manipulator, if any
Double 3	Telepresence robot	Self-balancing wheel	Various parts of SUTD Campus	Semi-autonomous	Travel across the site, communicating with others & viewing objects	Stereovision depth sensors, ultrasonic range finders,	25 x 23 x (119-150), 7.3 (adjustable height)	Adjustable neck

Task-based Test

Building	Zone	Building Component	Building element		Failure			Interaction	Effect	Severity[S]	Cause	Occurrence[O]	Current Preventive Measures	Detection[D]	RPN (S*O*D)	Recommended action
Building 2	*Level 6 Research Lab*	*Plan*	*28*	*Furniture layout (Office chairs)*	Accessibility		Robot has issues circumventing around obstacles	user getting robot to pass by walkway flanked by office chairs and robot prototype	Inefficiency due to extended recalibration and reorientation.	1	Lack of clearance	3	Nil	1	**3**	Consider different chair design
			29	*Furniture layout -Walkway*	Accessibility		robot has issues turning on the spot	user getting robot to turn around to go to next point of interest	Inefficiency due to extended recalibration and reorientation.	1	Lack of clearance	3	Nil	4	**12**	Adjust movable obstacles to provide clearance
			30	*Furniture layout- Walkway + trolley*	Accessibility		Robot got stuck between trolley and furniture	user getting robot to pass through obstacles in the walkway	Inefficiency due to extended recalibration and reorientation.	1	Lack of clearance	3	Nil	2	**6**	Adjust movable obstacles to provide clearance
											**Failure Count**		**3**	**Average RPN**		**7**
	L6 Cohort Classroom	*Interior*	*1, 2, 6*	*Furniture (table)*	Observability		The robot hits the table	The robot was attempting to move closer to the table to get a better look	Damage to robot and table	2	Sensors were unable to detect table as obstacle	3	nil	4	**24**	Provide markings around table to denote safe, accessible areas/ boundaries
			3		Observability	Manipulability	The robot falls flat onto the ground	The robot was unparking	Damage to robot and table, damage to robot casing and self-balancing required recalibration	5	The robot's self balancing measure kicked in upon unparking, causing a jerk which hit the table. The rebound was amplified by Internal gyroscopes causing rocking and the robot eventually lost balance as the robot rotated when sensors detected multiple obstacles around it. The disconnection of the user futher hampered any form of recovery.	3	nil	5	**75**	Provision of stable internet network. Provide markings around table to denote safe, accessible areas/ boundaries
		Electrical	*5*	*Electrical box*	Accessibility	Observability	Vibration and shaking of robot	Navigating over the covered ground power outlet	Minor damage to robot, Inefficiency due to extended recalibration and reorientation.	1	Box not flushed to ground surface, protruding slightly above the ground	2	nil	4	**8**	Warning markers on borders of electrical board
		Plan	*4*	*Layout*	*Activity*	Observability	Cannot find charging point	User tries to look for the charging point to dock	Inefficiency due to reorientation	2	User did not have information about surrounding whereabouts. Robot interface only provides information on location of charging point when it is in close proximity to charging dock.	3	nil	4	**24**	Provide visual/wayfinding markers to denote location of charging dock
											**Failure Count**		**6**	**Average RPN**		**21.8**

Diagnostic Test

Building	Zone	Building Component	Building element		Failure			Interaction	Effect	Severity[S]	Cause	Occurrence[O]	Current Preventive Measures	Detection[D]	RPN (S*O*D)	Recommended action
	Level 6 Research Lab	*Plan*	*1,5,13,27*	*Furniture layout*	Observability	Accessibility	Robot has issues circumventing around obstacles	User getting robot to turn around corner to change direction	Inefficiency due to extended recalibration and reorientation.	1	Lack of clearance	3	Nil	4	**12**	Adjust movable obstacles to provide clearance
			2,4,11,17	*Furniture layout*	Accessibility		Robot got stuck between loose items. Robot occassionally hits items when turning	User attempting to get robot to pass by tight gap	Minor damage to robot/objects	1	Lack of clearance	4	Nil	4	**16**	Adjust movable obstacles to provide clearance
			12	*Furniture (Storage box)*	Observability	Accessibility	Robot scrapes the edge of the box	Robot intended to go past the box and toward a designated corridor	Minor damage to robot	3	Lack of clearance, reflective edges of storage box	**2**	Nil	3	**18**	Adjust movable obstacles to provide clearance
			15	*Furniture layout*	Activity	Observability	Robot veers off from intended path by operator, Robot got lost and looped around the same area	User attempting to get robot back on track / intended path	Inefficiency due to extended recalibration and reorientation.	2	Too many obstacles in close proximity, causing inteference with sensors	4	Nil	4	**32**	Adjust movable obstacles to provide clearance
		Interior	*3*	*Furniture (transparent acrylic cylinder)*	Observability		Robot collides into transparent acrylic cylinder	Providing false option to user while navigating to desired location	Safety risk for robot	2	3D Vision camera cannot detect transparent surfaces, Sonar sensors could not detect the curved surface of the item	4	Nil	3	**24**	Improve obstacle detectability through additions
			17		Observability		Robot veered off course	Providing false option to user while navigating to desired location	Inefficiency due to extended recalibration and reorientation	1	Transparent object was first detected as accessible area before the robot moved too close to it and turned	3	Nil	3	**9**	Improve obstacle detectability through additions
			6	*Furniture (TV stand)*	Observability	Accessibility	Robot veers off from intended path by operator	User attempts to bypass TV stand obstacle	Inefficiency due to extended recalibration and reorientation	3	Linear and point objects are difficult to detect from a safe distance	2	Nil	4	**24**	Consider different design for the base stand
			9,18	*Furniture (Office chair)*	Observability	Accessibility	Robot's wheels got stuck between the chair legs	User getting robot to pass by irregularly-shaped obstacle	Inefficiency due to extended recalibration and reorientation	2	Obstacle is too low and out of sensor's range to be detected at a safe distance	4	Nil	4	**32**	Consider different chair design
			7,14,18,19,21,23,26		Observability		Robot veers off from intended path by operator	User getting robot to pass by irregularly-shaped obstacle	Inefficiency due to extended recalibration and reorientation	1	Obstacle is too low and out of sensor's range to be detected at a safe distance	4	Nil	3	**12**	Consider different chair design

Building 2		*Electrical*	*8,10,22,24,25*	*Electrical outlet cover*	Accessibility	Observability	Vibration and shaking of robot	Navigating over the covered ground power outlet	Minor damage to robot, Inefficiency due to extended recalibration and reorientation	2	Box with cable spacers that protrude above the ground	4	Nil	3	**24**	Demarcate no-go zones around electrical outlet by placing warning markers
											**Failure Count**		**27**	**Average RPN**		**19.5**
	L6 Transitional pace	*Exterior*	*1*	*Pathway (Corner between column and glass parapet)*	Accessibility		Robot stuck at tight corner between pillar and parapet	user attempting to get robot to pass by tight gap	Inefficiency due to extended recalibration and reorientation.	2	Lack of good field of vision. Wheels were too close to wall. Readings from sensors prevent robot from moving	2	Nil	1	**4**	Place ground markers to warn operator
		Interior	*2,5*	*Glass parapet*	Observability		False detection for 'clickable ground'	providing false option to user	Inefficiency due to extended recalibration and reorientation.	1	3D vision camera did not detect transparent glass as obstacle	5	Metal handle cap as detectable object	1	**5**	Change glass finish to fritted or frosted for glass near the ground to allow robot to allow glass surface as obstacle
			7		Observability		Detecting exterior overhung ledge as 'clickable ground' even with the glass parapet separating them	providing false option to user	Minor damage to robot and environment, Inefficiency due to extended recalibration and reorientation	2	3D vision camera cannot detect full glass surfaces well; does not register glass surface as obstacle	5	Metal handle cap as detectable object	1	**10**	Change glass finish to fritted or frosted for glass near the ground to allow robot to allow glass surface as obstacle
			9		Observability		Detecting exterior overhung ledge as 'clickable ground' even with the glass parapet separating them	providing false option to user	Collision with glass wall / obstacles	2	3D vision camera cannot detect full glass surfaces well; does not register glass surface as obstacle	5	Metal handle cap as detectable object	1	**10**	Change glass finish to fritted or frosted for glass near the ground to allow robot to allow glass surface as obstacle
			8	*Furniture (cantilevered table)*	Observability		False detection for 'clickable ground'	providing false option to user	Minor damage to robot and environment, Inefficiency due to extended recalibration and reorientation	2	Detectable area of the table for the robot is too limited	4	Nil	3	**24**	Place ground markers around obstacle footprint to avoid being too close to table
			12	*Lab door x4*	Observability		False positive for 'clickable ground'	providing false option to user	collision with glass wall / obstacles	1	3D vision camera cannot detect full glass surfaces well; does not register glass surface as obstacle	5	Door frame and handle as detectable objects	2	**10**	Change glass finish to fritted or frosted for glass near the ground to allow robot to allow glass surface as obstacle
			15	*Pathway*	Activity	Accessibility	Robot veers off course	attempting to bypass wall corner and dynamic obstacle by going in a bigger round	Inefficiency due to extended recalibration and reorientation.	1	false positives for obstacle sensors	2	Nil	4	**8**	Round sharp corners to increase accuracy in detection
			3	*Pathway (gap between parapet and pillar)*	Accessibility		Robot got trapped between pillar and parapet	user attempting to get robot to pass by tight gap	Rendered immobile	2	The width of pathway just fits robot's width, proximity sensor detects as insufficient clearance	5	Nil	4	**40**	Provide visual demarcations on safe distance away from the pillar
		Plan	*4*	*Pathway (gap between dustbins and pillar)*	Accessibility		Robot got stuck between dustbin and pillar	user attempting to get robot to pass by tight gap ; Sensor constant feedback	Inefficiency due to extended recalibration and reorientation.	2	The width of pathway just fits robot's width, proximity sensor detects as insufficient clearance	5	Nil	3	**30**	Reposition dustbin
		Structure	*6*	*Staircase (going down)*	Activity	Observability	Enters no-go zone due to misalignment of cliff sensor	robot falls over if no human intervention occurred	Safety risk	5	The robot was too close to the stairs. Sensors were misaligned when robot tried to rotate and move forward	2	Thin metal strip along step	2	**20**	Avoid stairs / Change path / have ground markers for hazard detection to inform robot of no-go zone
		Exterior	10	*Parapet*	Activity	Observability	Robot wheels hit against sloping concrete interface	robot attempting to go straight	Damage to wheels	2	There is tendency for lower part of the parapet to be detected as traversible area	1	Metal railing as detectable object	2	**4**	Change parapet interface with ground / have ground markers on edge to avoid being too close to parapet
			11	*Structure (Wall)*	Activity	Observability	Robot got trapped along wall surface	attempting to backtrack / turn while wheels were flush against wall	damage to wheels, inability for robot to turn and reverse and take alternate path	3	Wheel face were too close to wall surface. Readings from sensors prevent robot from moving	2	Nil	3	**18**	Place ground markers on edge to avoid being too close to wall
			14		Activity		Collision against people	avoiding multiple obstacles simultaneously, erroneous decision taken by robot algorithm	Collision with glass wall / obstacles	3	obstacle avoidance mechanism conflicted with presence of multiple obstacles and took disadvantageous route	1	Nil	4	**12**	Emergency stop system for robot during collisions
		Exterior	*13*	*Fixture (Door stopper)*	Observability		Collision with door stopper	attempting to go past obstacle	Damage to robot	2	Door stopper is too low in height to be detectable by robot sensors	1	Nil	5	**10**	Place ground markers to avoid being too near to stopper
											**Failure Count**		**15**	**Average RPN**		**13.8**
Building 2,3	*L1 Campus Centre*	*Interior*	*1*	*Furniture (Table)*	Accessibility		Robot hits the edge of table leg	Robot was navigating to its next designated location	Slight damage to robot and table	2	Obstacle is too low and out of sensor's range to be detected at a safe distance	**2**	Nil	3	**12**	Place ground markers around obstacle footprint to avoid no-go zone
			2	*Metal railing*	Observability		Disorientation for robot	Robot attempting to avoid detected obstacles	Inefficiency due to extended recalibration and reorientation	2	Sensors did not detect thin reflective surfaces as obstacle	2	Metal handle cap as detectable object	2	**8**	Increase obstacle's side surface area for better detection / improve robot sensor range
			3	*Glass door (with fritting)*	Observability		False positive for 'clickable ground'	providing false option to user	Possible collision with glass wall / obstacles	1	3D vision camera cannot detect full glass surfaces well; fritting is viewed against white background; not enough contrast	3	Fritting patterns which should help detectability	3	**9**	Use tinted glass / change finishing of glass surface
		Exterior	*7*	*Glass door*	Observability		False positive for 'clickable ground'	Providing false option to user	Possible collision with glass wall / obstacles	1	3D vision camera cannot detect full glass surfaces well; does not register glass surface as obstacle	5	Door frame and handle as detectable objects	4	**20**	Use tinted glass / change finishing of glass surface
		Interior	*4*	*Furniture (retractable ribbon barrier belt)*	Observability		Robot collides into the ribbon belt	Robot was navigating to its next designated location	Possible damage to robot and surroundings	2	Difficulty of robot in detecting linear objects at particular height	3	Nil	4	**24**	Consider alternative types of queue barriers that allows easy detectability
			5	*Furniture (Permanent Installation)*	Observability		Robot's head hit against installation part	Robot was navigating to its next designated location	Minor damage to robot and installation	2	Installation part was at a height out of robot's sensing range	2	Nil	4	**16**	Place ground markers around obstacle footprint to alert robot of no-go zone
			6	*Furniture (Metal rubbish bin)*	Observability		Robot's wheel lightly hit the bin	Robot was navigating to its next designated location	Minor damage to robot and bin	1	Robot was too near to the bin. Upon rotation, the sensors did not pick up the reflective surface well.	3	Nil	4	**12**	Change finishing of bin
			8	*Furniture (Metal signage stand)*	Accessibility		Robot got stuck	Robot was navigating to its next designated location	Inefficiency due to extended recalibration and reorientation	2	Metal surfaces caused robot's sensors to recalibrate over a period of time	3	Nil	2	**12**	Remove signage where possible. Use a different signage stand design
			9	*Furniture (Sofa)*	Activity	Observability	Robot veered onto no-go zone	False positive option for 'clickable area'. Robot was navigating towards no-go zone.	Contribute to wear and tear of robot. Disorientation for operator. Possible damage to robot and surroundings	4	Sofa cushion was flushed to ground surface. The change in material + soft material of cushion can cause imbalance problems for self-balancing wheel.	2	Yellow tape marking out the boundary	4	**32**	Place ground markers to demarcate no-go zones. Add cushioning around edges to prevent robot entry
			10	*Finishes (Change in flooring)*	Accessibility		Slight shaking of robot	Robot was navigating across floor of differing material	Contribute to wear and tear of robot. Disorientation for operator	2	The transition between the change of material was not levelled, resulting in slight change in elevation	2	Nil	3	**12**	Ensure that transitions should be levelled

		Services	*11*	*Cable casing*	Accessibility	Observability	Robot gets disoriented from original direction	Robot to overcome cable casing	Eventually hits object while trying to self-balance, Damage to robot and environment. Robot goes into a loop while trying to self-balance	4	Casing was located next to an obstacle. Robot's direction was shifted suddenly and hits before obstacle avoidance features could kick in.	3	Nil	4	**48**	Rewire trunking/casing to an area away from the metal railing. Otherwise, introduce a gentler gradient / flatter curvature
			12, 14	*Cables on floor*	Accessibility		Shaking of robot	Robot overcoming cable obstacle	Contribute to wear and tear of robot. Disorientation for operator	3	Loose cables acting as bumps for the robot	5	Nil	3	**45**	Add trunking/casing with a gentler gradient / flatter curvature
			13	*Electrical point cover*	Accessibility		Shaking of robot	Robot overcoming cable obstacle	Contribute to wear and tear of robot. Disorientation for operator	1	Cover protude from the ground, acting as bumps for the robot	3	Nil	4	**12**	Alter cover design to minimise level change
											**Failure Count**		**14**	**Average RPN**		**18.7**
